# A Comprehensive Study Utilizing QSAR, Virtual Screening, Molecular Docking, Molecular Dynamics, and MM/GBSA Analyses Reveals Natural Diterpenoids as Promising Caspase-1 Inhibitors

**DOI:** 10.3390/molecules31162894

**Published:** 2026-08-19

**Authors:** Yusuf Şeflekçi, Alper Yılmaz, Abdulilah Ece

**Affiliations:** 1 Department of Molecular Biology and Genetics, Faculty of Art and Sciences, Yildiz Technical University, Istanbul 34220, Türkiye; 2 Department of Molecular Biology and Genetics, Faculty of Engineering and Natural Sciences, Biruni University, Istanbul 34015, Türkiye; 3 Department of Medical Biochemistry, Faculty of Medicine, Biruni University, Istanbul 34015, Türkiye

**Keywords:** caspase-1, inflammation, QSAR, natural products, molecular dynamics, MM/GBSA

## Abstract

Caspase-1 is a crucial inflammatory cysteine protease that facilitates the maturation of pro-inflammatory cytokines such as interleukin-1β and interleukin-18, making it a significant therapeutic target for inflammatory diseases. However, existing caspase-1 inhibitors often face challenges like toxicity and suboptimal drug-like properties, underscoring the need for new inhibitors. This study employed an integrated computational strategy, combining quantitative structure–activity relationship (QSAR) modeling and application of this validated model to a large natural product database followed by molecular docking, rigorous binding free energy analysis and extended molecular dynamics simulations. Initially, a dataset of 185 caspase-1 inhibitors with experimentally reported pKi values (ranging from 4.05 to 9.24) was used to construct a QSAR model using Partial Least Squares (PLS) regression. The PLS-based QSAR model was developed with 18 descriptors out of 5799 calculated descriptors for each compound and 10 latent variables, demonstrating strong statistical performance with R^2^ values of 0.870 and 0.838 for the training and test sets, respectively, and leave-one-out cross-validation coefficient Q^2^_LOO_ and 5-fold cross-validation (Q^2^_5-fold_) values of 0.819 and 0.814, respectively. Y-randomization tests further confirmed the model’s robustness, as the randomized models exhibited significantly lower statistical parameters than the original model. The validated QSAR model was applied to 276,518 natural products in the LOTUS database. Subsequent molecular docking, Molecular Mechanics/General Born Surface Area (MM/GBSA) scoring, and Pan-Assay INterference Compounds (PAINS) and Chemical Frequent Hitter (ChemFH) filtering identified 14 candidate compounds, which were further evaluated using 300 ns molecular dynamics simulations. Among these, four natural products (LTS0162325, LTS0221286, LTS0016840, and LTS0070407) showed the most stable binding behavior and maintained persistent interactions with key catalytic and substrate-binding residues of caspase-1 in a mimicked physiological condition. Overall, this study highlights natural diterpenoids and coumarin glycosides as promising scaffolds for caspase-1 inhibition and demonstrates that integrating QSAR modeling with structure-based approaches provides an efficient strategy for discovering potential anti-inflammatory drug candidates.

## 1. Introduction

Caspases (cysteine-dependent aspartate-specific proteases) play essential roles in physiological processes including inflammation and apoptosis. Caspase-1 (interleukin-1β-converting enzyme) is an inflammatory caspase that is responsible for the production of pro-inflammatory cytokines such as IL-1β and IL-18, which recruit inflammatory cells to infection sites [1]. It is also involved in the maturation of IL-33, known as a T helper type 2 (TH2)-associated cytokine [2]. Caspase-1 is activated by inflammasome complexes, which are essential during innate immune responses, resulting in cleavage of pro-IL-1β and pro-IL-18 into active forms [3,4]. Additionally, activation of caspase-1 stimulates gasdermin-mediated pyroptosis, which increases intracellular content and cytokine release [5]. Mature IL-1β participates in several physiological processes, including inflammation, cell proliferation, differentiation, and pyroptosis [6,7]. Moreover, the overproduction of IL-1β has been linked to the emergence of various conditions, including inflammatory and autoimmune disorders, arthritic diseases, septic shock, and neurodegenerative illnesses such as Alzheimer’s and Parkinson’s [8]. Thus, targeting caspase-1 to block IL-1β and IL-18 presents a promising approach for treating various diseases [9], as inhibition of caspase-1 has been indicated to be beneficial in various cytokine-mediated disorders including rheumatoid arthritis, osteoarthritis, atherosclerosis, septic shock, and inflammatory bowel syndrome [10,11]. Accordingly, caspase-1 has been recognized as a key regulator of inflammation and an important therapeutic target for the discovery and development of anti-inflammatory drugs [12].

Currently, several peptides, peptidomimetics and small molecules have been designed as caspase-1 inhibitors for the treatment of caspase-1-related disorders [11]. Moreover, several anti-IL-1β agents like IL-1Ra (anakinra) [13] or anti-IL-1β antibodies (canakinumab) [14] have also been utilized successfully in many inflammatory and autoimmune disorders. However, IL-1Ra is highly costly, needs repeated injections, and can cause allergic reactions. Pralnacasan and VX-765 are highly effective caspase-1 inhibitors and have been utilized in clinical trials aimed at treating rheumatoid arthritis and psoriasis. Although Pralnacasan indicated promising results in phase II trials, liver abnormalities were observed in animal studies [15]. Therefore, identifying novel caspase-1 inhibitors is essential for the development of safer and more effective therapeutic agents for the treatment of inflammatory diseases.

One of the modern strategies to reduce the cost and time associated with drug discovery and to overcome experimental challenges is the use of computational approaches, including quantitative structure–activity relationship (QSAR) modeling, virtual screening, molecular docking, and molecular dynamics simulations. These computational methods have become essential tools for the identification and development of novel drug candidates [16,17]. QSAR correlates molecular descriptors of compounds with their measured biological activities to generate mathematical models that allow biological activity prediction of new compounds [18]. Thus, it has emerged as one of the effective and valuable tools for drug design [18,19]. Prior to experimental validation, QSAR is primarily applied to estimate the biological potencies of unknown compounds. This approach can support the processes of lead discovery and optimization when designing new molecules [20].

The combination of QSAR with molecular docking and molecular dynamics simulations will further improve the reliability of the results [21]. Several QSAR-based studies have been performed for the identification of new caspase-1 inhibitors in the literature. A multitarget QSAR–MLP approach has recently identified several promising dual caspase-1/TNF-α inhibitors for broad-spectrum anti-inflammatory therapy [22]. In another study, the authors combined QSAR and molecular docking to identify the structural features that enhance caspase-1 inhibition [23]. Natural products (NPs) are characterized by a wide range of chemical structures and biological functions, making them an important resource for drug development [24]. Therefore, in this study, a QSAR model was constructed using 5799 molecular descriptors and applied to screen 276,518 natural products from the LOTUS database to identify novel caspase-1 inhibitors.

## 2. Materials and Methods

### 2.1. Data Collection and Preparation

A total of 185 compounds against caspase-1 were obtained from the ChEMBL database (https://www.ebi.ac.uk/chembl/, accessed on 18 November 2025) with biological activity data (pKi values) from a similar inhibition assay [25]. These structures were then converted into 3D structures and energy minimized using Cresset Flare™ V11.0 (Cresset^®^, Litlington, Cambridgeshire, UK; https://cresset-group.com/software/flare/, accessed on 23 July 2026).

### 2.2. Calculation of Molecular Descriptors

alvaDesc v3.0 (Alvascience, Lecco, Italy; https://www.alvascience.com/alvadesc/, accessed on 23 July 2026) [26] was used to calculate up to 5799 descriptors (from 0- to 3-dimensional descriptors) for each compound. Prior to training the model, a feature selection procedure was performed. Accordingly, all constant variables, variables with only one unique value, and variables that had at least one sample with a missing value or exhibited autocorrelation greater than 0.95 were eliminated. The remaining 1753 molecular descriptors were utilized for QSAR model generation.

### 2.3. QSAR Model Building and Validation

Prior to the QSAR model building, the dataset was manually divided into training (136 compounds) and test (49 compounds) sets through an activity-stratified sampling strategy. This approach was employed to ensure a homogeneous distribution of pKi values and prevent bias in model validation. Then, a QSAR model was generated by alvaModel v3.0 (Alvascience, Lecco, Italy; https://www.alvascience.com/alvamodel/, accessed on 23 July 2026) [27] based on the Partial Least Squares (PLS) methods using 10 latent variables (LV = 10). The optimal number of latent variables is automatically selected using the SIMPLS algorithm [28]. This method relies on transforming a huge number of correlated characteristics into a small number of uncorrelated variables known as latent variables [29]. This method is also powerful for using a large number of variables [30].

To prevent model overparameterization and reduce the risk of chance correlations, the number of descriptors included in the final QSAR model was limited based on the compound-to-descriptor ratio. As a generally accepted rule of thumb, at least 5 compounds are required for one descriptor; we set this ratio to 10:1. Thus, the final QSAR model development was restricted to a maximum of eighteen variables (180 compounds). An approximate ratio of 10:1 was applied as the model complexity control criterion. Accordingly, based on the size of the training set, the final PLS-QSAR model was constructed using 18 descriptors selected from the filtered descriptor pool [31].

The constructed QSAR was validated to test the model’s accuracy, stability, and predictive ability. QSAR model was assessed using the following statistical metrics: the coefficient of determination (R^2^), root mean square error (RMSE) and mean absolute error (MAE) on the training and test sets. Also, the leave-one-out cross-validation coefficient (Q^2^_LOO_) and coefficient of determination of 5-fold cross-validation (Q^2^_5-FOLD_) were utilized to check robustness.

The applicability domain (AD) of the QSAR model was evaluated utilizing the leverage approach (Hat matrix) implemented in alvaModel. AD is utilized to find structural and response outliers from the test and training set, respectively [32,33]. A Williams’ plot was generated by plotting leverage values against standardized residuals. The warning leverage value (h*) was calculated as follows:

(1) $$h^{*}=3\frac{\left(p+1\right)}{n}$$

where p is the number of descriptors and *n* is the number of training compounds [34].

A Y-randomization test comprising 100-response permutations was conducted to evaluate the reliability and robustness of the developed QSAR model and to eliminate the possibility of any speculative correlations. This test was essential to demonstrate the model’s predictive accuracy regarding the activities of the training set compounds [35,36].

### 2.4. QSAR-Based Virtual Screening and Activity Prediction

The generated QSAR model was applied to the natural product database LOTUS (https://lotus.naturalproducts.net) dataset (accessed on: 20 December 2025) for predicting the pKi of natural compounds. Compounds in the database were first filtered through Lipinski’s rule of five (Ro5). Then, alvaRunner was used to predict the activity values of each compound (https://www.alvascience.com/alvarunner/, accessed on 23 July 2026). Compounds with pKi values equal to or greater than 9 were selected for the molecular docking analysis.

### 2.5. Molecular Docking

Molecular Docking analysis was performed using Cresset Flare™ V11.0 (Cresset^®^, Litlington, Cambridgeshire, UK; https://cresset-group.com/software/flare/). For this purpose, the crystal structure of caspase-1 (PDB ID: 6PZP; 1.94 Å Resolution) was retrieved from the Protein Data Bank (https://www.rcsb.org). Then, the structure was optimized by removing water molecules, adding missing H atoms, and modifying side chain protonation states. After preparation, docking scores of the compounds (both native and natural compounds dataset) were calculated using binding sites of the crystal structure.

### 2.6. MM/GBSA Calculation

The binding free energies of the docked compounds and native ligand were estimated by the Molecular Mechanics/General Born Surface Area (MM/GBSA) method [37] using Cresset’s Flare™ Pro+ software. The solvent model was selected as Implicit GBn2, and both the protein and ligand were minimized [38]. The binding energy calculation was performed using the following equation:

(2) $$G_{bind}=E_{bond}+E_{elec}+E_{VdW}+G_{pol}+G_{non-pol}-TS$$

All terms in the equation are defined as follows:

*G_bind_* = binding free energy of receptor and ligand, *E_bond_* = bond energy, *E_elec_* = electrostatic energy, *E_VdW_* = van der Waals energy, *G_pol_* = polar energy, *G_non-pol_* = difference between non-polar and polar energy, and *TS* = conformational entropy.

### 2.7. In Silico PAINS and ChemFH Filter

Compounds with docking scores lower than −8 kcal/mol and MM/GBSA binding free energy values lower than −50 kcal/mol were selected, followed by filtering using the Pan-Assay INterference Compounds (PAINS) and Chemical Frequent Hitter (ChemFH), an integrated online platform (https://chemfh.scbdd.com, accessed on 20 January 2026) to detect frequent false positives.

### 2.8. Molecular Dynamics Simulation

Compounds filtered through ChemFH were used for the molecular dynamics analysis based on two criteria: (i) those with MM/GBSA binding free energy values lower than −58 kcal/mol, and (ii) compounds exhibiting docking scores lower than −13 kcal/mol and MM/GBSA binding free energy values lower than −50 kcal/mol. Also, molecular dynamics analysis was performed for the apo and co-crystallized ligand-bound form of 6PZP. Molecular dynamics (MD) simulation was carried out using Cresset’s Flare™ Pro+ software to assess the conformational changes and stability of the ligands in complex with the protein. Also, after neutralizing the overall charge of the system, solvent ionic strength was set as 0.15 M. MD simulation was run for 300 ns in transferable intermolecular potential 3P (TIP3P) solvent using the AMBER force field by selecting a truncated octahedron solvent box shape. Then, root mean square deviation (RMSD) and root mean square fluctuation (RMSF) plots were computed to analyze the structural stability of the ligand–protein complex.

### 2.9. Trajectory-Based MM/GBSA Calculations

Following molecular dynamics simulations, trajectory-based MM/GBSA calculations were performed using Cresset Flare™ V11.0 (Cresset^®^, Litlington, Cambridgeshire, UK; https://cresset-group.com/software/flare/) to estimate the binding free energies of the 14 selected compounds and the native ligand over the simulation trajectories. To identify the most promising candidates, the trajectory-based MM/GBSA results were evaluated together with the overall stability of the protein–ligand complexes, as assessed by the RMSD profiles throughout the simulations. Thus, lead compounds were selected based on the combined interpretation of dynamic stability and binding free energy rather than on trajectory-based MM/GBSA values alone.

### 2.10. Binding Interaction Determination

Following the molecular dynamics analysis, the interacting residues of the potential compounds with the 6PZP protein were identified. For this purpose, K-means clustering analysis was performed between backbone atoms. Then, the cluster with the highest fraction value was selected to analyze the interaction sites of each potential compound.

## 3. Results and Discussion

### 3.1. QSAR Model Development and Validation

**Figure 1:**
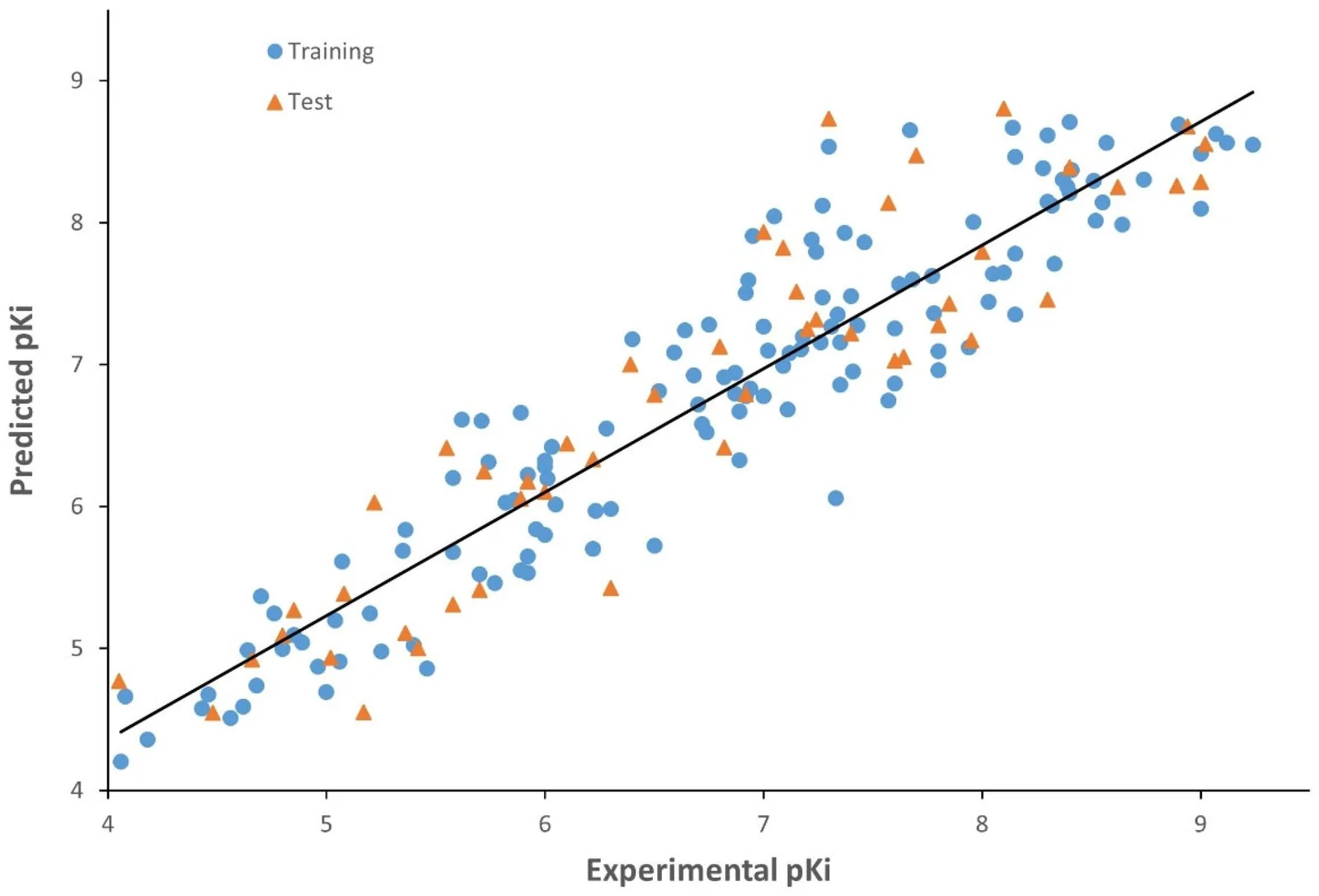
Regression plot of experimental versus predicted pKi values for the training and test sets of caspase-1 inhibitors used in the development of the QSAR model.

The developed PLS-based QSAR model demonstrated strong predictive performance and robustness. The plot shows the experimental activity (pKi) versus the predicted activity, providing information about how well the model was trained and how well it predicts the activity of the external test set (Figure 1). The experimental and predicted activity values and their residual values were presented in Table S1. The activity value distribution is also given in Figure S1.

The coefficient of determination (R^2^) values for the training and test datasets were 0.870 and 0.838, respectively, exceeding the commonly accepted threshold (R^2^ > 0.6) for reliable QSAR models [35], and demonstrating a strong correlation between the predicted and experimental pKi values. In addition, leave-one-out cross-validation coefficient (LOO) and coefficient of determination of 5-fold cross-validation (Q^2^_5-FOLD_) were calculated as 0.819 and 0.814, respectively, indicating the robustness of the developed QSAR model [39,40]. Furthermore, the possibility of a chance correlation was also evaluated by performing a Y-randomization test. The randomized models produced significantly lower statistical parameters, with mean R^2^, Q^2^_LOO_, and Q^2^_5-fold_ values of 0.063, 0.001, and 0.002, respectively, and maximum values of 0.177, 0.036, and 0.057, respectively. These results clearly demonstrate that the predictive performance of the developed QSAR model is not due to chance correlation (Table 1).

**Figure 2:**
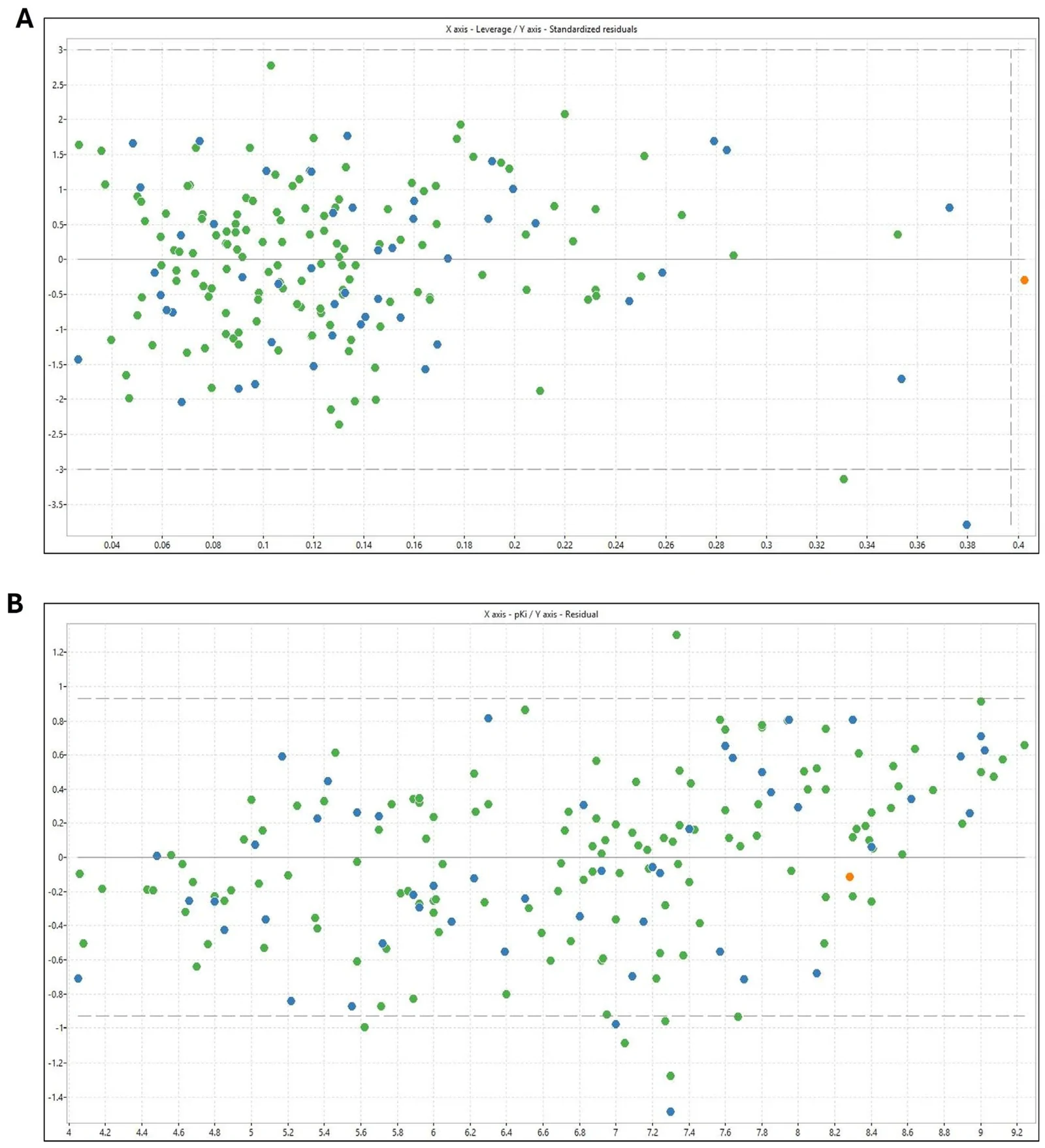
(**A**) Williams’ plot showing standardized residuals versus leverage values for the QSAR model. Dashed horizontal lines indicate the ±3 standardized residual limits. (**B**) Plot of residuals versus experimental pKi values used to evaluate the distribution of prediction errors. Green dots represent compounds in the training set, whereas blue dots represent compounds in the external test set.

The applicability domain (AD) of the developed QSAR model was evaluated using Williams’ plot, which represents standardized residuals versus leverage values. As shown in Figure 2, most of the compounds were located within the acceptable leverage threshold and within the standardized residual limits of ±3 [41], indicating the absence of significant response outliers. Most compounds in both the training and test sets were located within the applicability domain of the model. These results indicate that the developed QSAR model has a well-defined applicability domain and reliable predictive capability.

Finally, after evaluating all those statistical metrics above, the following QSAR equation was obtained:

$$\begin{array}{cc} {pK}_{i}=6.795 & -0.195\times VE1_{X}-0.245\times Chi_{Dt}-0.059\times MATS7p\\ & -0.108\times SM13_{EA\left(ed\right)}+0.268\times SM10_{AEA\left(bo\right)}\\ & +0.044\times DISPm+0.082\times WiA_{Coulomb}\\ & +0.045\times TDB03u+0.254\times TDB07e\\ & +0.171\times TDB08r+\;0.147\times RDF110s\\ & -0.059\times Mor10e-0.399\times nArCO\\ & -0.202\times B05\left[O-O\right]+0.710\times F05\left[O-O\right]\\ & -0.927\times RPCG+0.389\times DLS_{02}\\ & -0.179\times CATS3D_{05_{DL}} \end{array}$$

### 3.2. Descriptor Interpretation

While SM10_AEA(bo), DISPm, WiA_Coulomb, TDB03u, RDF110s, F05[O-O], and DLS_02 descriptors correlate positively to the inhibitory activity, VE1_X, Chi_Dt, MATS7p, SM13_EA(ed), TDB07e, TDB08r, Mor10e, nArCO, B05[O-O], RPCG and CATS3D_05_DL descriptors correlate negatively to the inhibitory activity (Table 2). Calculated values of selected molecular descriptors for all compounds are illustrated in Table S2.

Among the descriptors related to edge adjacency indices, SM10_AEA(bo) exhibited a positive contribution to the inhibitory activity, indicating that enhanced bond connectivity patterns and local molecular topology favor biological activity. In contrast, SM13_EA(ed) showed a negative contribution, suggesting that higher-order edge adjacency features or excessive structural complexity may adversely affect the inhibitory activity. These descriptors belong to the class of edge adjacency indices that describe the adjacency relationships between bonds in a molecular graph and encode structural information related to bond connectivity patterns [42]. They capture structural information related to bond connectivity patterns, branching, and local molecular topology, which can influence biological activity.

The geometrical descriptor DISPm exhibited a positive contribution, indicating that increased molecular size and spatial distribution favor inhibitory activity. This suggests that ligands with flexible substituents and extended conformations may better adapt to the binding pocket of caspase-1, facilitating stronger interactions with catalytic and surrounding residues [43].

Electrostatic and 3D structural descriptors, including WiA_Coulomb and RDF110s, further highlight the importance of the charge distribution and spatial arrangement of atoms. The positive contribution of WiA_Coulomb indicates that favorable electrostatic interactions between atom pairs enhance the binding affinity, which is consistent with the polar nature of the caspase-1 active site [44,45]. Similarly, RDF110s reflect the radial distribution of atomic mass, suggesting that the steric and spatial organization of functional groups plays a crucial role in stabilizing ligand binding [46].

The presence of 3D autocorrelation descriptors (TDB03u, TDB07e, and TDB08r) indicates that both short- and long-range interactions contribute to the activity. While TDB03u captures local structural organization, TDB07e and TDB08r encode long-range electrostatic and steric effects, suggesting that an optimal spatial arrangement across the entire molecule is required for effective inhibition [47,48,49].

In contrast, negatively contributing descriptors, such as VE1_X, Chi_Dt, and MATS7p, are related to molecular topology and polarizability [42,50]. Their negative contributions suggest that excessive structural complexity or unfavorable distribution of polarizable atoms may reduce the activity. Similarly, the negative contribution of nArCO indicates that the presence of aromatic carbonyl groups may be detrimental, possibly because of suboptimal interactions within the binding pocket or steric hindrance [42].

The inclusion of atom pair descriptors, particularly F05[O–O] (positive) and B05[O–O] (negative), highlights the importance of the oxygen atom distribution within the molecular framework [42]. This suggests that specific spatial arrangements of oxygen-containing functional groups can enhance hydrogen bonding and electrostatic interactions with key residues, such as HIS237, GLY238, and GLN283.

Furthermore, the CATS3D_05_DL descriptor, which encodes donor–lipophilic interactions at a defined spatial distance [51], negatively contributed to activity, indicating that certain donor–lipophilic arrangements may not favor optimal binding within the caspase-1 active site.

In addition, the presence of the 3D-MoRSE descriptor, Mor10e, highlights the importance of the spatial distribution of electronegativity within the molecular framework. As this descriptor is weighted by Sanderson electronegativity, it reflects how electron-rich and electron-deficient regions are arranged in three-dimensional space [52,53]. This suggests that proper alignment of electrostatic potential within the ligand is crucial for forming favorable interactions with the caspase-1 active site.

The inclusion of the charge descriptor RPCG further emphasizes the role of electrostatic interactions in determining biological activity. RPCG accounts for the relatively positive charge distribution within the molecule, indicating that regions capable of generating a positive electrostatic potential may enhance binding through interactions with negatively polarized residues or hydrogen bonding [54,55,56].

Moreover, the drug-likeness descriptor DLS_02 indicates that compounds that satisfy specific physicochemical constraints tend to exhibit improved inhibitory activity. This suggests that, beyond binding affinity, overall molecular properties, such as hydrogen bonding capacity, flexibility, and ring count, contribute to the effectiveness of caspase-1 inhibitors. The presence of this descriptor in the model highlights the importance of balancing potency and drug-like characteristics during compound optimization [57].

**Table 2:** Definition of the descriptors in the QSAR model.

Descriptor	Description	Class	Contribution
SM10_AEA(bo)	Spectral moment of order 10 from augmented edge adjacency mat. weighted by bond order	2D	Positive
DISPm	Displacement value/weighted by mass		Positive
WiA_Coulomb	Average Wiener-like index from Coulomb matrix	3D	Positive
TDB03u	3D topological distance-based descriptors—lag 3 unweighted	2D	Positive
RDF110s	Radial distribution function-110/weighted by relative I-state	3D	Positive
F05[O-O]	Frequency of O–O at topological distance 5	2D	Positive
DLS_02	Modified drug-like score from Oprea et al. [57] (6 rules)	3D	Positive
VE1_X	Coefficient sum of the last eigenvector (absolute values) from chi matrix	3D	Negative
Chi_Dt	Randic-like index from detour matrix	2D	Negative
MATS7p	Moran autocorrelation of lag 7 weighted by polarizability	3D	Negative
SM13_EA(ed)	Spectral moment of order 13 from edge adjacency mat. weighted by edge degree	2D	Negative
TDB07e	3D topological distance-based descriptors—lag 7 weighted by Sanderson electronegativity	2D	Negative
TDB08r	3D topological distance-based descriptors—lag 8 weighted by covalent radius	2D	Negative
Mor10e	Signal 10/weighted by Sanderson electronegativity	3D	Negative
nArCO	Number of ketones (aromatic)	2D	Negative
B05[O-O]	Presence/absence of O–O at topological distance 5	2D	Negative
RPCG	Relative positive charge	3D	Negative
CATS3D_05_DL	CATS3D Donor–Lipophilic BIN 05 (5.000–6.000 Å)	2D	Negative

### 3.3. QSAR-Based Virtual Screening

The validated QSAR model was then applied to screen the LOTUS natural product database comprising 276,518 compounds. Initially, to ensure drug-likeness and oral bioavailability, the dataset was filtered according to Lipinski’s rule of five criteria, which reduced the library to 138,124 compounds. The refined dataset was subsequently subjected to activity prediction using the developed QSAR model, and the pKi values were calculated for each compound. Compounds exhibiting predicted pKi values equal to or greater than 9 were selected as potential inhibitors. The use of a QSAR-based filtering step prior to structure-based methods provides a significant advantage by reducing the computational cost and focusing downstream analyses on compounds with a high probability of activity. This hierarchical screening strategy is consistent with the best practices in computer-aided drug design, where ligand-based methods are used for initial filtering, followed by structure-based refinement [58]. This criterion resulted in 5733 compounds which were further subjected to molecular docking.

### 3.4. Molecular Docking and MM/GBSA Calculation

**Figure 3:**
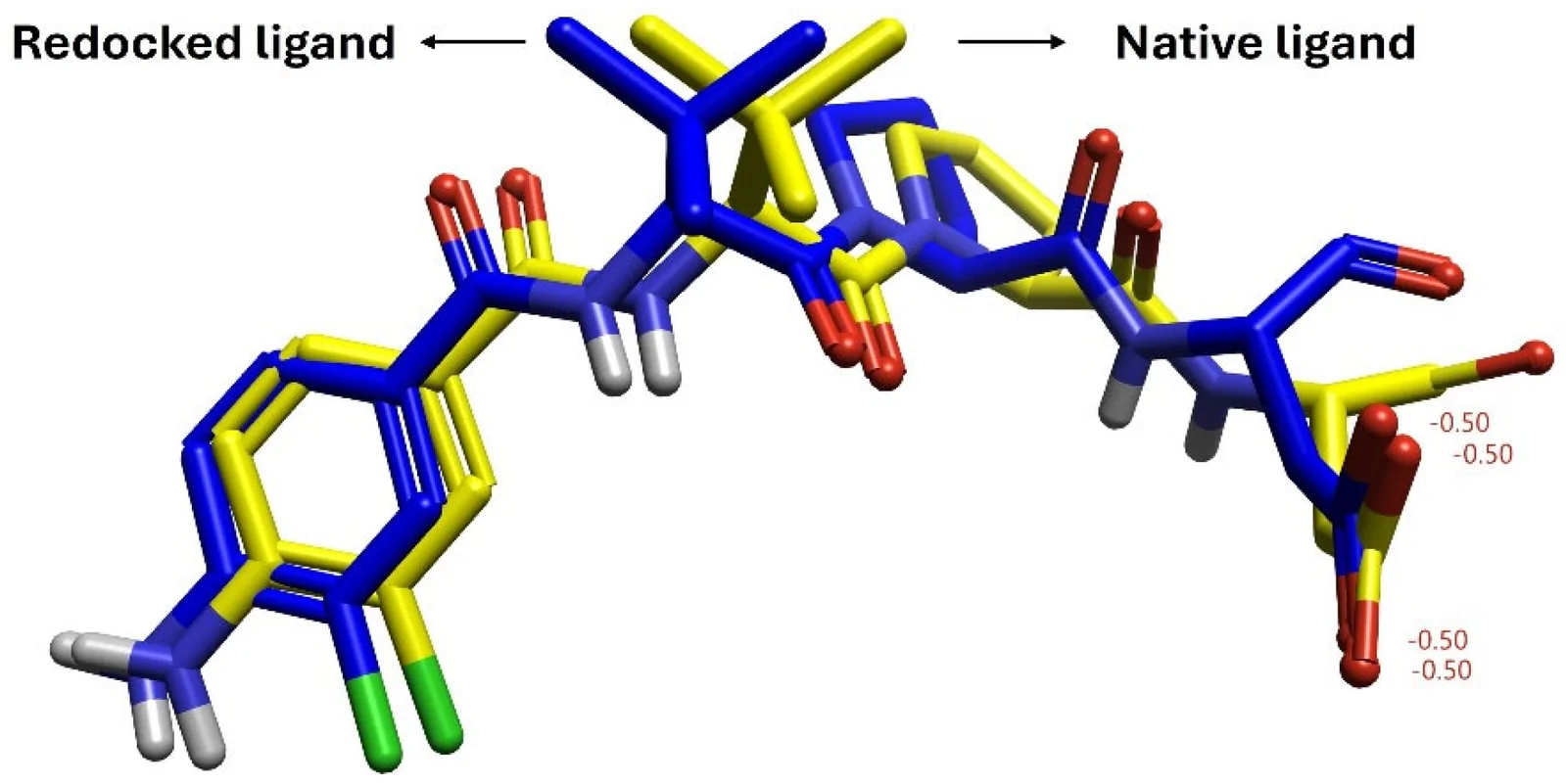
Internal validation of the docking protocol through redocking analysis.

The docking protocol was validated by redocking the co-crystallized ligand at the active/native ligand site of the caspase-1 protein. Then, the root mean square deviation (RMSD) of the redocked ligand was calculated as 0.907 Å using DockRMSD (https://aideepmed.com/DockRMSD/, accessed on 5 February 2026) (Figure 3). This result confirms the reliability of the docking procedure, as values below 2.0 Å are generally considered acceptable for reproducing experimental binding poses [59,60].

The computed docking score of the redocked native ligand was found to be −11.724 (kcal/mol). While molecular docking offers important preliminary insights into ligand–receptor interactions and binding affinities, it is important to recognize its limitations. Docking scoring functions frequently depend on simplified energy models that neglect dynamic changes in protein conformation and the presence of explicit solvent molecules, which can lead to inaccurate predictions of binding energies and ligand poses [61]. To address these issues, this study utilized MM/GBSA free energy calculations and molecular dynamics simulations, providing a more realistic evaluation of ligand binding stability and affinity [62,63].

The MM/GBSA calculation for the native ligand yielded a value of −58.42 kcal/mol. Then, both docking and MM/GBSA scores were used as reference points for evaluating the screened compounds. Subsequently, all compounds selected from QSAR-based virtual screening (5733 compounds) were docked into the protein’s active site. Compounds with docking scores lower than −8 kcal/mol and MM/GBSA binding free energy values lower than −50 kcal/mol were chosen (Table 3).

### 3.5. In Silico PAINS and ChemFH Filtering

The integration of PAINS and ChemFH filtering significantly enhanced the reliability of the screening pipeline by reducing the risk of false positives. PAINS are known to produce nonspecific interactions and false-positive results in high-throughput screening [64], whereas frequent hitter prediction tools, such as ChemFH, are specifically designed to identify such artifacts. Therefore, the application of these filters is a critical step in improving the reliability of computational drug discovery workflows [65].

Following docking and MM/GBSA-based filtering, 80 compounds satisfied the PAINS criteria. Among these, nine compounds were excluded because of unfavorable structural features, resulting in 71 compounds that were subsequently evaluated using the ChemFH filter. After ChemFH screening, 67 compounds were classified as “pass.” From this refined set, the top 14 compounds exhibiting better MM/GBSA compared to the native ligand were selected, and compounds that have considerably better docking scores than the native ligand were also added to this set for further molecular dynamics (MD) simulations. The docking scores, MM/GBSA energies, and ChemFH filtering results for the selected compounds are indicated in Table 3.

### 3.6. Molecular Dynamics

**Figure 4:**
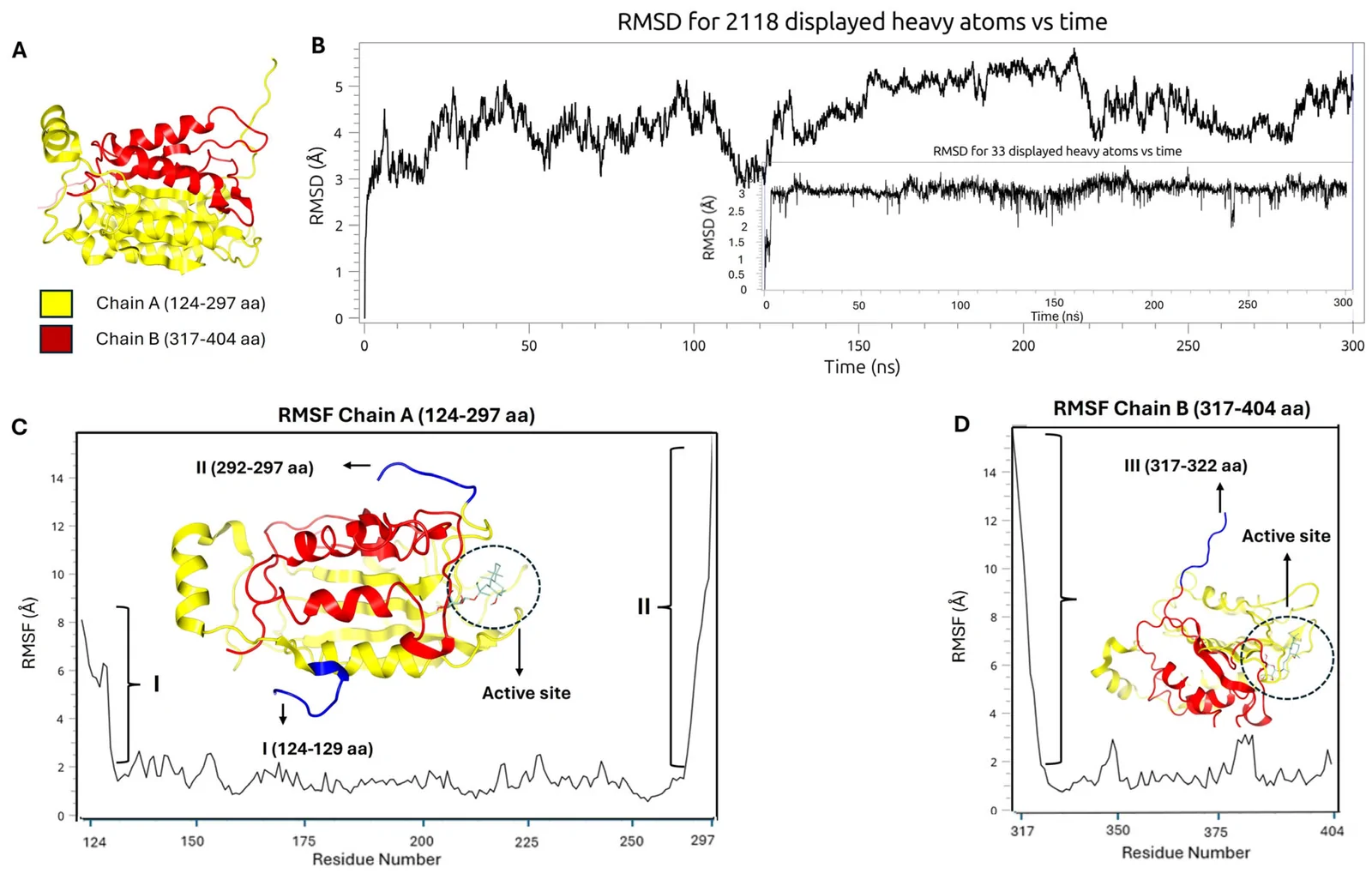
Molecular dynamics analysis of LTS0162325–caspase-1. (**A**) Chain-colored representation of the caspase-1 dimer, with chain A (residues 124–297) shown in yellow and chain B (residues 317–404) shown in red. (**B**) RMSD plot of complete LTS0162325–caspase-1 over the course of a 300 ns MD simulation. The inset shows the RMSD of the ligand heavy atoms over the same simulation period. (**C**) RMSF plot of chain A (residues 124–297) of the LTS0162325-caspase-1 complex. Region I (residues 124–129) and region II (residues 292–297) indicate highly fluctuating regions highlighted in blue. (**D**) RMSF plot of chain B (residues 317–404) of the LTS0162325-caspase-1 complex. Region III (residues 317–322) indicate highly fluctuating regions highlighted in blue.

**Figure 5:**
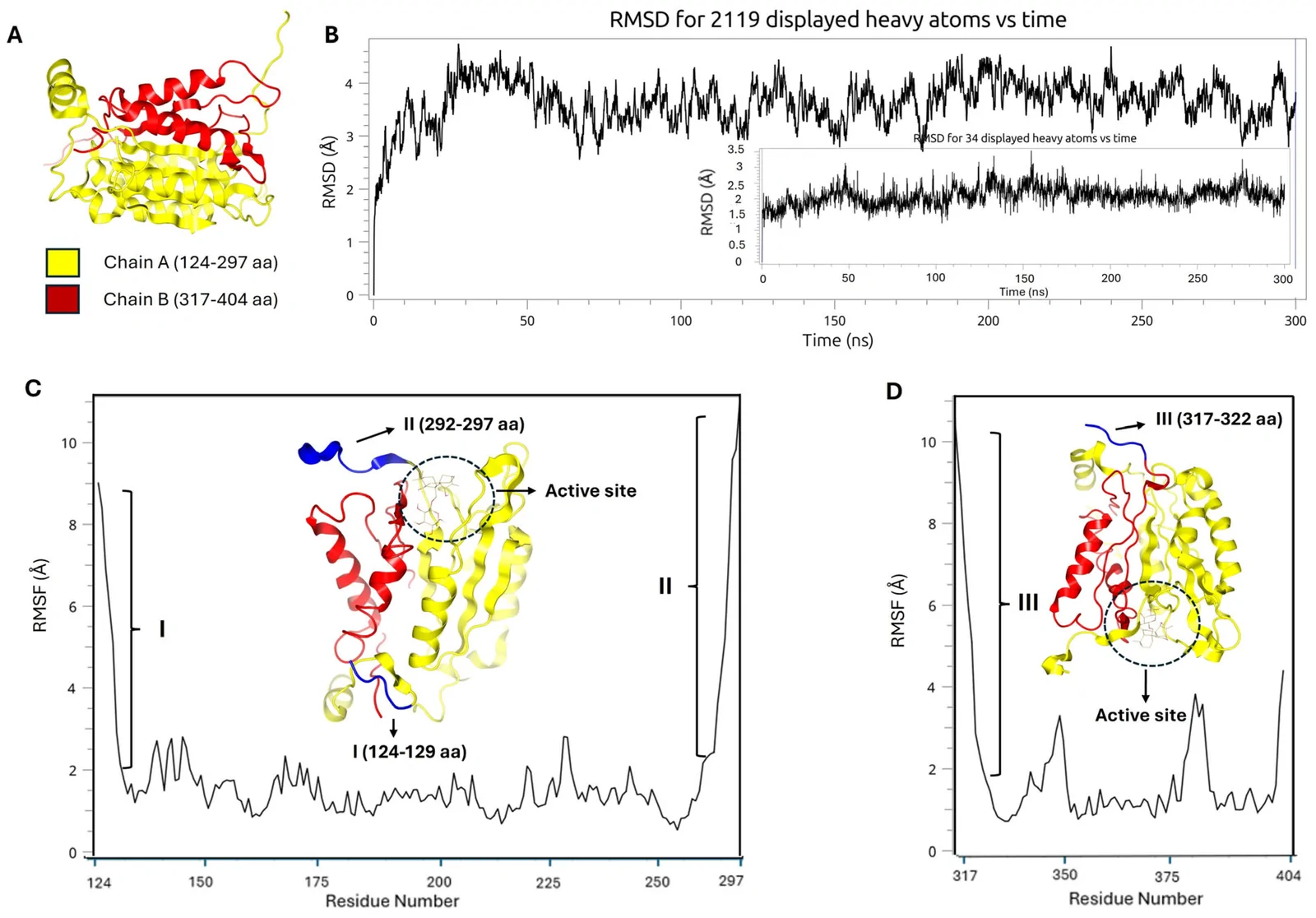
Molecular dynamics analysis of LTS0221286–caspase-1. (**A**) Chain-colored representation of the caspase-1 dimer, with chain A (residues 124–297) shown in yellow and chain B (residues 317–404) shown in red. (**B**) RMSD plot of complete LTS0221286–caspase-1 over the course of a 300 ns MD simulation. The inset shows the RMSD of the ligand heavy atoms over the same simulation period. (**C**) RMSF plot of chain A (residues 124–297) of the LTS0221286-caspase-1 complex. Region I (residues 124–129) and region II (residues 292–297) indicate highly fluctuating regions highlighted in blue. (**D**) RMSF plot of chain B (residues 317–404) of the LTS0221286-caspase-1 complex. Region III (residues 317–322) indicate highly fluctuating regions highlighted in blue.

**Figure 6:**
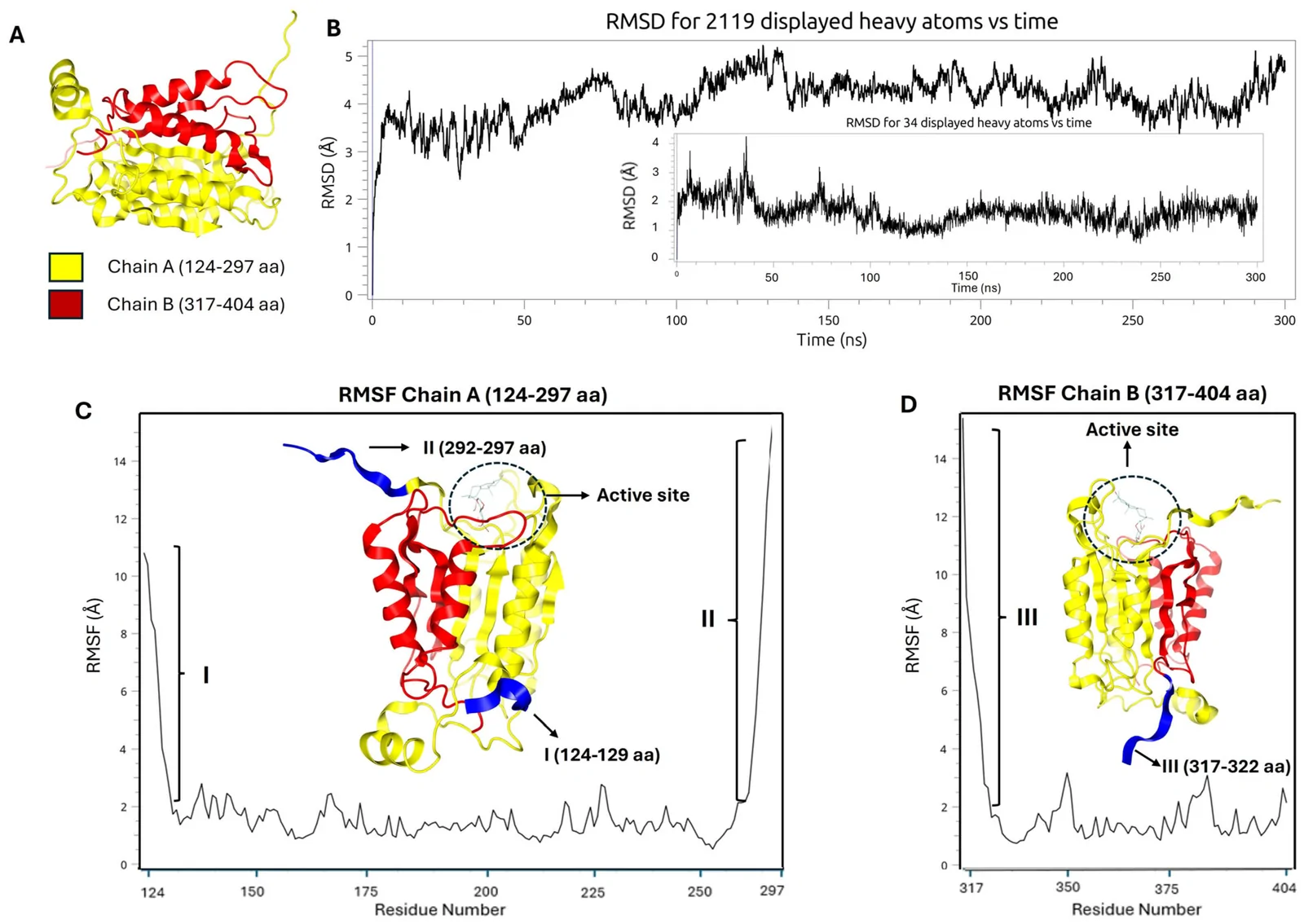
Molecular dynamics analysis of LTS0016840–caspase-1. (**A**) Chain-colored representation of the caspase-1 dimer, with chain A (residues 124–297) shown in yellow and chain B (residues 317–404) shown in red. (**B**) RMSD plot of complete LTS0016840–caspase-1 over the course of a 300 ns MD simulation. The inset shows the RMSD of the ligand heavy atoms over the same simulation period. (**C**) RMSF plot of chain A (residues 124–297) of the LTS0016840–caspase-1 complex. Region I (residues 124–129) and region II (residues 292–297) indicate highly fluctuating regions highlighted in blue. (**D**) RMSF plot of chain B (residues 317–404) of the LTS0016840–caspase-1 complex. Region III (residues 317–322) indicate highly fluctuating regions highlighted in blue.

**Figure 7:**
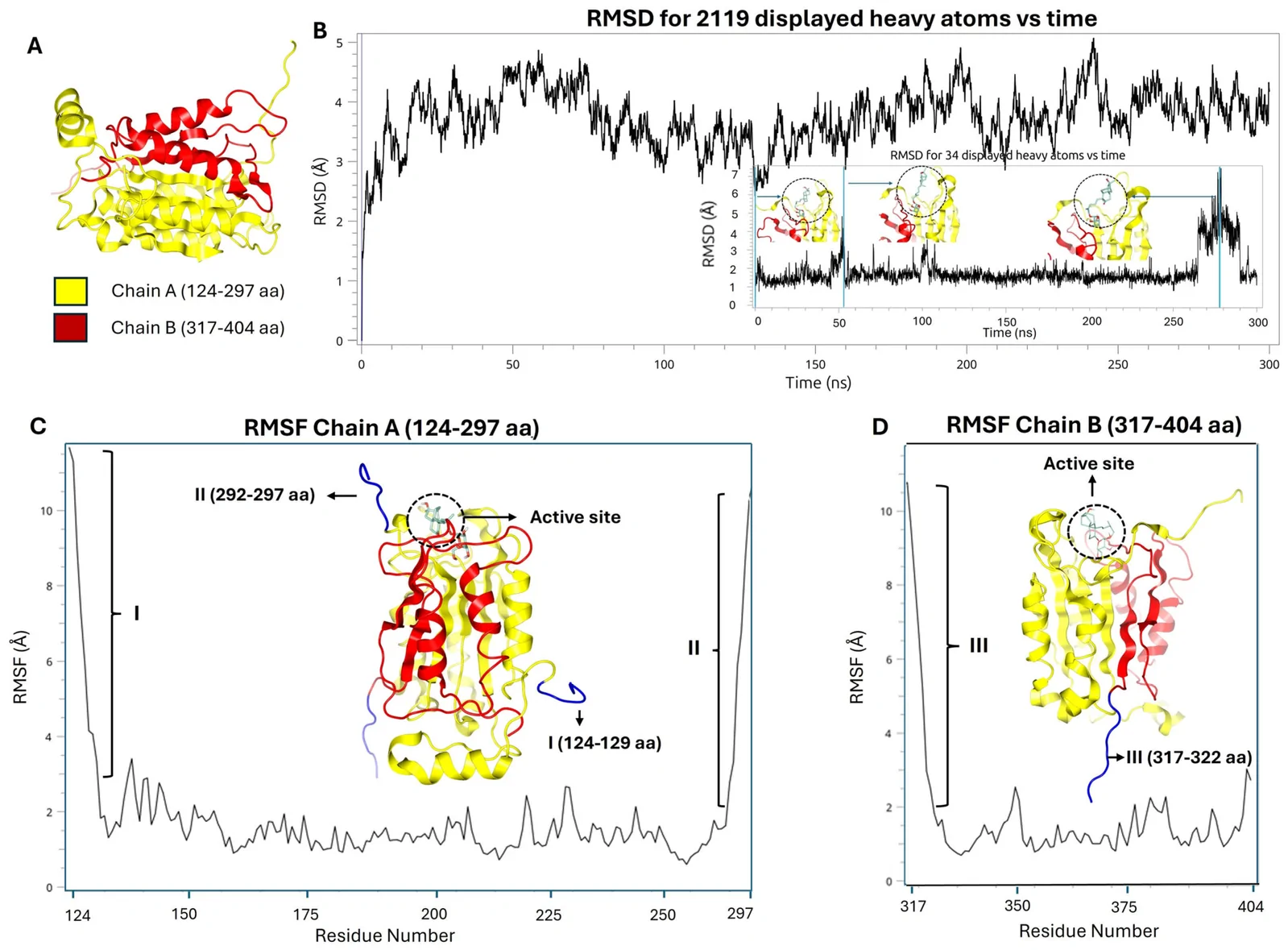
Molecular dynamics analysis of LTS0070407–caspase-1. (**A**) Chain-colored representation of the caspase-1 dimer, with chain A (residues 124–297) shown in yellow and chain B (residues 317-404) shown in red. (**B**) RMSD plot of complete LTS0070407–caspase-1 over the course of a 300 ns MD simulation. The inset shows the RMSD of the ligand heavy atoms over the same simulation period. (**C**) RMSF plot of chain A (residues 124–297) of the LTS0070407-caspase-1 complex. Region I (residues 124–129) and region II (residues 292–297) indicate highly fluctuating regions highlighted in blue. (**D**) RMSF plot of chain B (residues 317–404) of the LTS0070407-caspase-1 complex. Region III (residues 317–322) indicate highly fluctuating regions highlighted in blue.

Molecular dynamics simulations typically simulate the motions of atoms over time, which helps predict the interaction of ligands within physiological environments [66,67]. The RMSD and RMSF analyses of the apo and co-crystallized ligand-bound form of 6PZP are indicated in Figure S2. Among the fourteen analyzed complexes, four compounds (LOTUS ID: LTS0162325, LTS0221286, LTS0016840, and LTS0070407) exhibited the most stable behavior based on the RMSD profile calculated for the complete protein–ligand complex. The dimeric structure of caspase-1 was visualized by chain, with chain A (residues 124–297) and chain B (residues 317–404) to provide chain-based RMSF analysis (Figure 4A, Figure 5A, Figure 6A and Figure 7A). Although small fluctuations were observed till the end of simulation time, RMSD analysis revealed that the LTS0162325-6PZP complex exhibited continuous backbone fluctuations of 3.0–5.5 Å without reaching a tight plateau (Figure 4B), whereas the corresponding ligand RMSD (Figure 4B, inset) remained largely confined within a narrow 3.0–3.5 Å range throughout the trajectory, indicating a stable binding pose despite the overall backbone flexibility. The LTS0221286-6PZP complex, by contrast, reached a stable plateau after approximately 25 ns and remained within a 3.5–4.5 Å range during the simulation (Figure 5B). The corresponding ligand RMSD (Figure 5B, inset) remained consistently within a narrow 1.5–2.5 Å range throughout the trajectory, indicating that LTS0221286 preserved a stable binding orientation within the active site despite the moderate backbone fluctuations observed for the protein. The LTS0016840-6PZP complex stabilized after approximately 50 ns, fluctuating within a 3.5–5.0 Å range for the remainder of the trajectory (Figure 6B). The corresponding ligand RMSD (Figure 6B, inset) remained largely confined to approximately 1.0–2.2 Å throughout the simulation, with only a brief increase during the early equilibration phase. This behavior indicates that LTS0016840 maintained a stable binding orientation within the active site without evidence of ligand dissociation. The LTS0070407-6PZP complex reached equilibrium within the first 50 ns and remained the most stable among the four complexes, fluctuating within a narrow 3.0–4.5 Å range throughout the 300 ns simulation (Figure 7B). The corresponding ligand RMSD (Figure 7B, inset) remained low for most of the simulation but exhibited a noticeable increase during the final stage of the trajectory. Visual inspection of the MD trajectory revealed that this increase was not associated with ligand dissociation from the binding pocket; instead, LTS0070407 retained its binding pose and key protein–ligand interactions, indicating that the elevated ligand RMSD most likely resulted from a local conformational reorientation within the binding site rather than loss of binding stability. The RMSF plot showed that many of the amino acid residues displayed fluctuations below 2 Å, except for several loop regions which were located away from the active site. Specifically, the major fluctuation peaks corresponded to regions I and II of chain A and region III of chain B. Importantly, residues surrounding the ligand-binding region exhibited comparatively low fluctuations, indicating the maintenance of a relatively stable binding site during the simulations [68] (Figure 4C,D, Figure 5C,D, Figure 6C,D and Figure 7C,D). These findings support the hypothesis that the identified compounds form stable complexes with caspase-1 and are capable of maintaining interactions under dynamic conditions.

### 3.7. Trajectory-Based MM/GBSA Analysis

As shown in Table 3, the trajectory-based MM/GBSA binding free energies ranged from −14.37 to −39.81 kcal/mol for the screened compounds, whereas the native ligand exhibited the most favorable binding free energy (−46.20 kcal/mol). Among the 14 candidates, four compounds—LTS0162325 (−39.81 kcal/mol), LTS0221286 (−36.23 kcal/mol), LTS0016840 (−35.05 kcal/mol), and LTS0070407 (−34.33 kcal/mol)—displayed the most favorable trajectory-based binding free energies, closely approaching that of the native ligand. Notably, these four compounds also exhibited the lowest and most stable ligand RMSD profiles throughout the 300 ns simulation, with values consistently maintained within 1–3.5 Å after the initial equilibration phase and without evidence of ligand dissociation or major pose reorientation. This convergence between the energetic (MM/GBSA) and structural (RMSD) metrics provides strong, independent support for the stability of the predicted binding modes of these four compounds within the active site.

### 3.8. Binding Interaction Analysis

**Figure 8:**
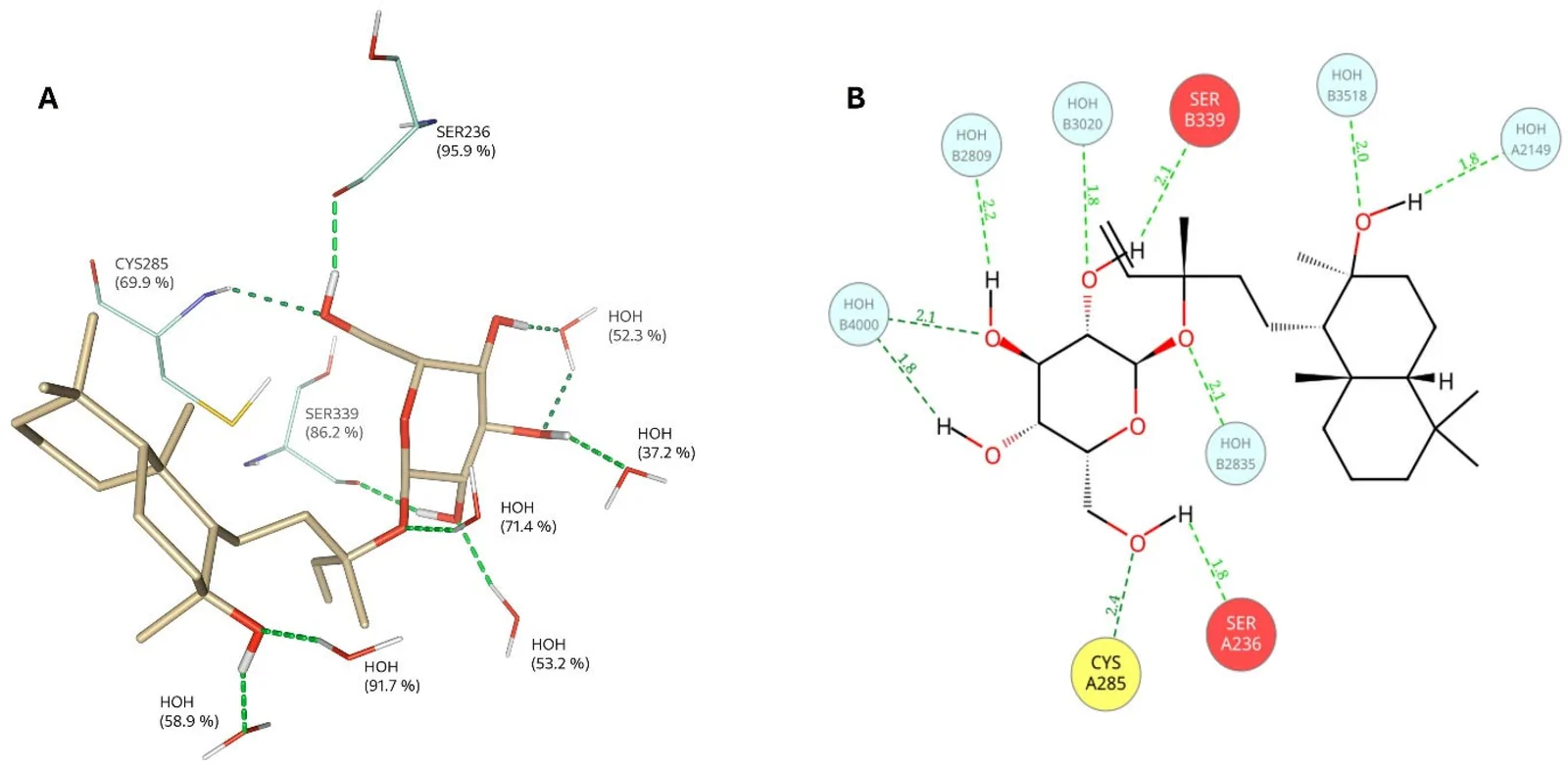
(**A**) The 3D and (**B**) 2D binding interaction map of LTS0162325 within the active site of caspase-1. Hydrogen bonds and sulfur–lone pairs are represented as green and purple, respectively.

Interaction analysis of the LTS0162325-6PZP complex revealed that the ligand formed a highly persistent interaction network dominated by hydrogen bonds with residues surrounding the catalytic pocket. The hydrogen bonds (green dashed lines) with SER236 (95.9%), SER339 (86.2%), and CYS285 (69.9%), together with several water-mediated hydrogen bonds that remained present for 37.2–91.7% of the simulation time, further stabilized the ligand within the binding site. The exceptionally high interaction occupancy with SER236, together with direct interaction with the catalytic residue CYS285, indicates that the ligand remained tightly anchored within the active site during the simulation. The additional contribution of SER339 and solvent-mediated interactions further enhanced complex stability, supporting the rapid RMSD convergence and favorable binding free energy observed for this compound (Figure 8A,B).

**Figure 9:**
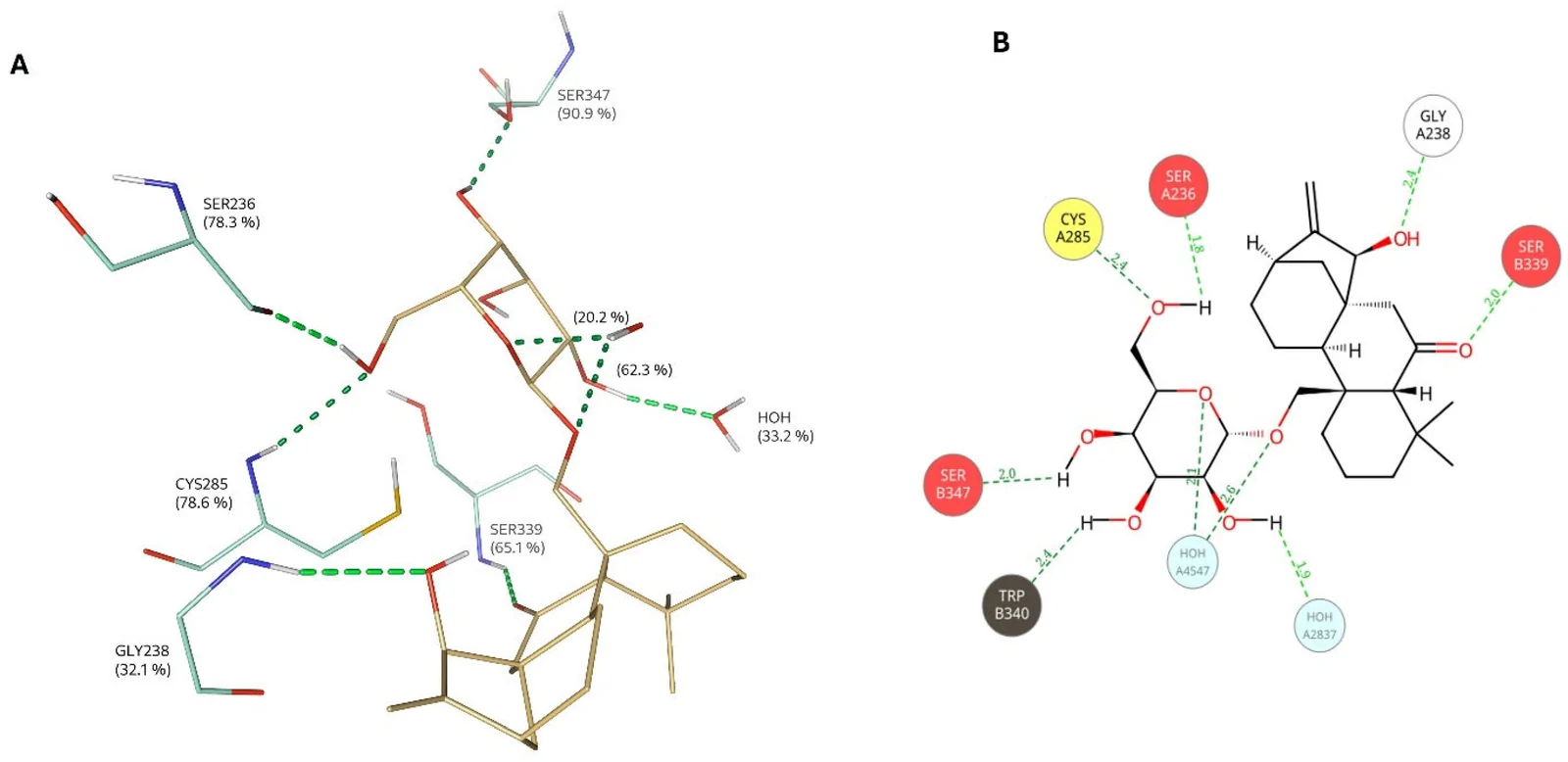
(**A**) The 3D and (**B**) 2D binding interaction map of LTS0221286 within the active site of caspase-1. Hydrogen bonds and sulfur–lone pairs are represented as green and purple, respectively.

Interaction analysis of the LTS0221286–6PZP complex demonstrated that the ligand established stable hydrogen-bonding interactions (green dashed lines) with several key residues involved in substrate recognition and catalysis. Persistent interactions were observed with SER347 (90.9%), CYS285 (78.6%), SER236 (78.3%), and SER339 (65.1%), whereas GLY238 showed a lower interaction occupancy (32.1%). Water-mediated hydrogen bonds additionally contributed to stabilization of the complex throughout the simulation. Importantly, the interaction with the catalytic residue CYS285, together with multiple hydrogen bonds involving SER236, SER339, and SER347, suggests that the ligand adopts a stable orientation within the active-site cavity. These interaction patterns are consistent with the stable MD trajectory and favorable trajectory-based MM/GBSA binding energy calculated for LTS0221286 (Figure 9A,B).

**Figure 10:**
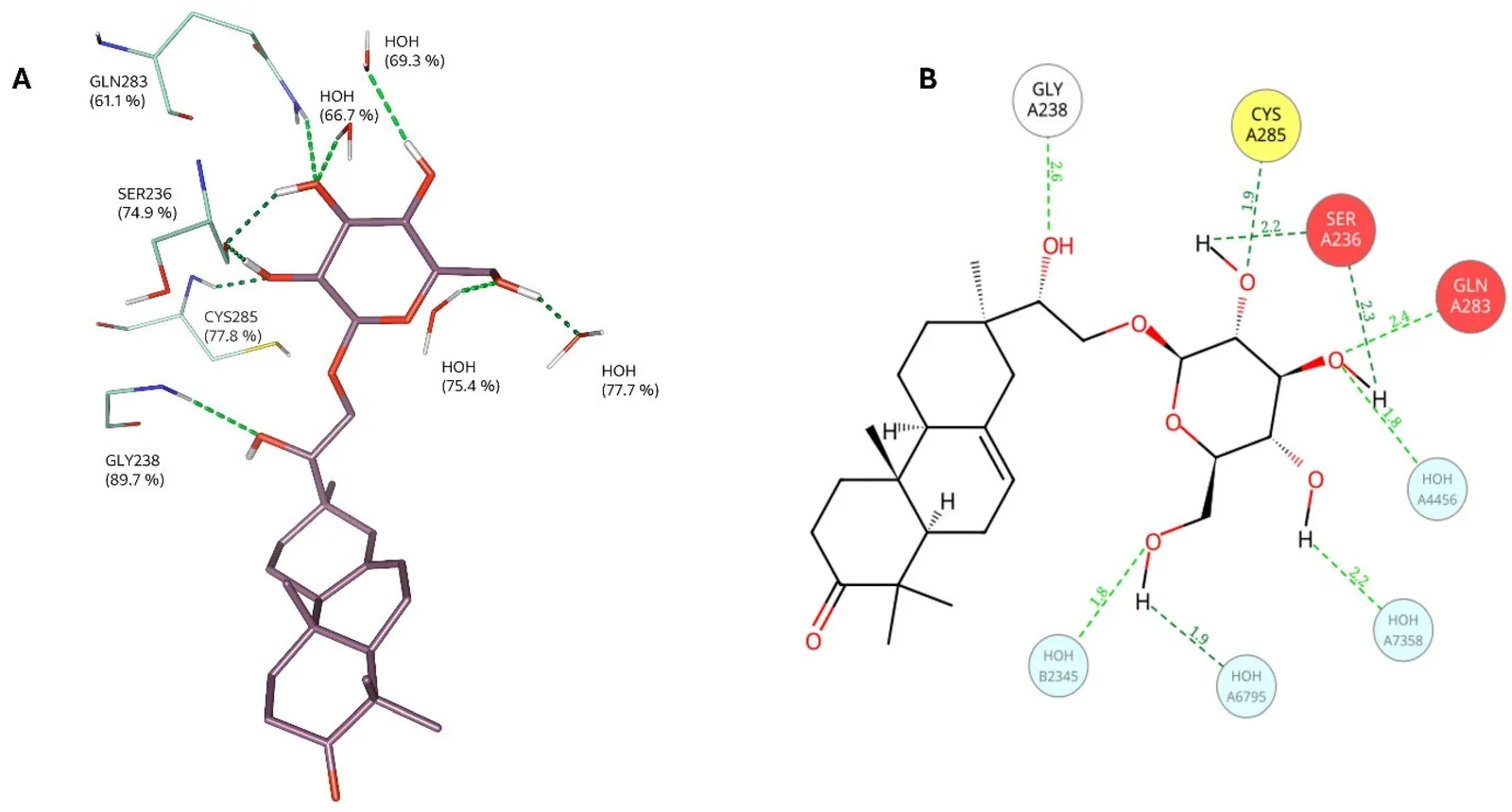
(**A**) The 3D and (**B**) 2D binding interaction map of LTS0016840 within the active site of caspase-1. Hydrogen bonds and sulfur–lone pairs are represented as green and purple, respectively.

Interaction analysis of the LTS0016840–6PZP complex revealed that the ligand established stable hydrogen-bonding interactions with several residues located within the catalytic pocket of caspase-1. LTS0016840 exhibited hydrogen bonds (green dashed lines) with GLY238 (89.7%), CYS285 (77.8%), SER236 (74.9%), and GLN283 (61.1%) throughout the simulation. In addition, multiple water-mediated hydrogen bonds were maintained for 66.7–77.7% of the simulation time, further stabilizing the ligand within the binding pocket. Notably, interaction with the catalytic residue CYS285 suggests that LTS0016840 is appropriately oriented toward the catalytic center, whereas simultaneous interactions with SER236, GLY238, and GLN283 contribute to stabilization of the substrate-binding region. Together, these findings support the stable binding mode observed during molecular dynamics simulations and are consistent with the favorable trajectory-based MM/GBSA binding energy obtained for this compound (Figure 10A,B).

**Figure 11:**
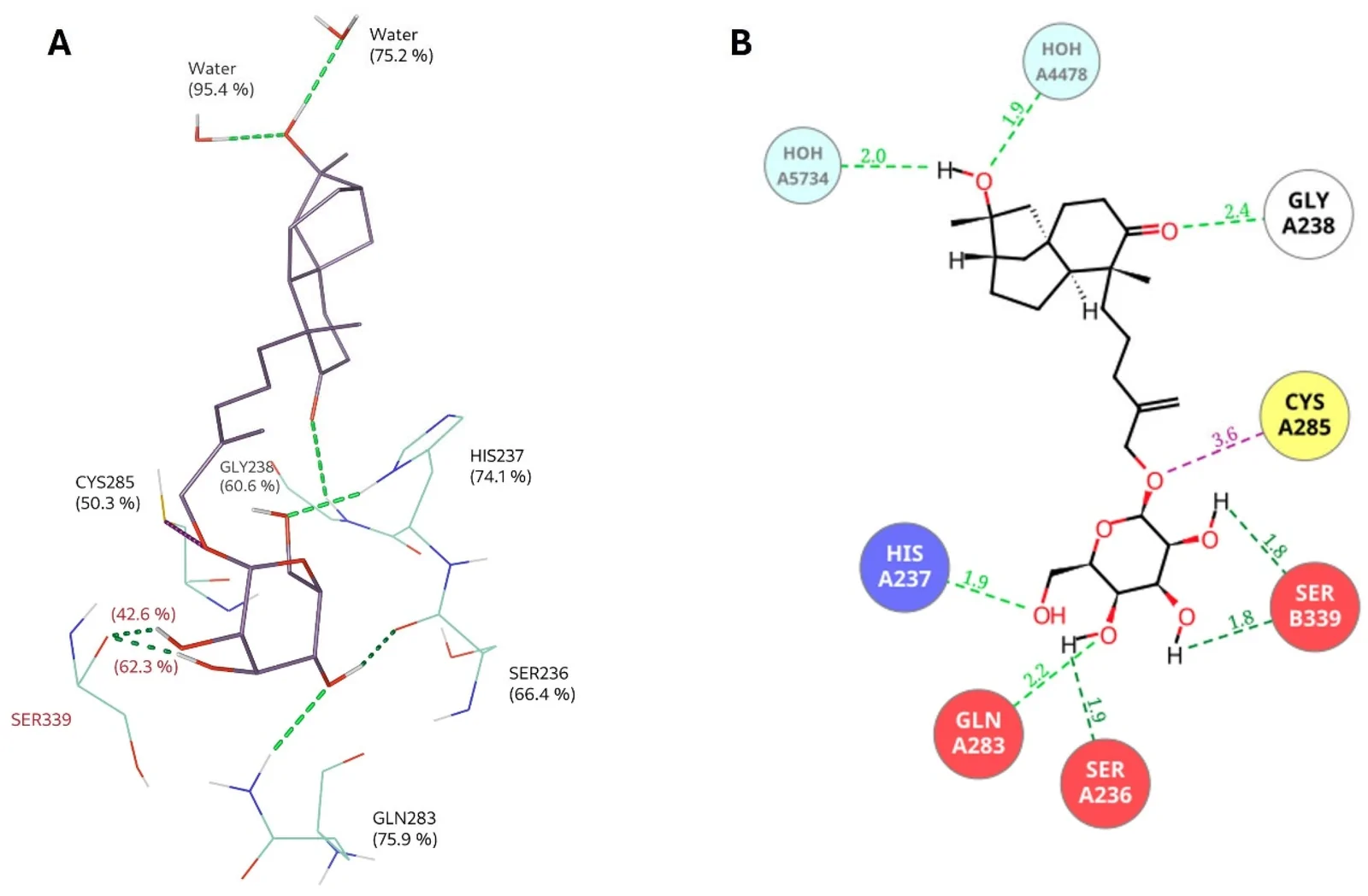
(**A**) The 3D and (**B**) 2D binding interaction map of LTS0070407 within the active site of caspase-1. Hydrogen bonds and sulfur–lone pairs are represented as green and purple, respectively.

Detailed interaction analysis revealed that the identified compounds established stable and persistent interactions with the key catalytic and substrate-binding residues of caspase-1. Interaction analysis of the LTS0070407–6PZP complex over the 300 ns MD trajectory revealed hydrogen bond and stabilizing interactions within the active site of caspase-1. LTS0070407 exhibited strong hydrogen bonding interactions (green dashed lines) with GLN283, HIS237, SER236, SER339, and GLY238, which were maintained for 75.9%, 74.1%, 66.4%, 62.3% and 60.6%, respectively, throughout the total simulation. Also, water-mediated hydrogen bonds were observed for 75.2% and 95.4% of the total simulation time. Notably, interaction with the catalytic residue CYS285 via sulfur–lone pair interaction (purple dashed line) suggests potential inhibition of enzymatic activity by directly targeting the catalytic site (Figure 11A,B).

These findings are consistent with the known structural organization of caspase-1, where HIS237, GLY238, and CYS285 form the catalytic core and residues such as ARG179, GLN283, ARG341, and SER347 contribute to the formation of binding pocket, which stabilizes substrate binding within the active site [69,70]. The ability of the identified compounds to interact with these key residues suggests a potential mechanism of inhibition through the disruption of substrate binding and catalytic activity.

### 3.9. Lead Compounds

**Figure 12:**
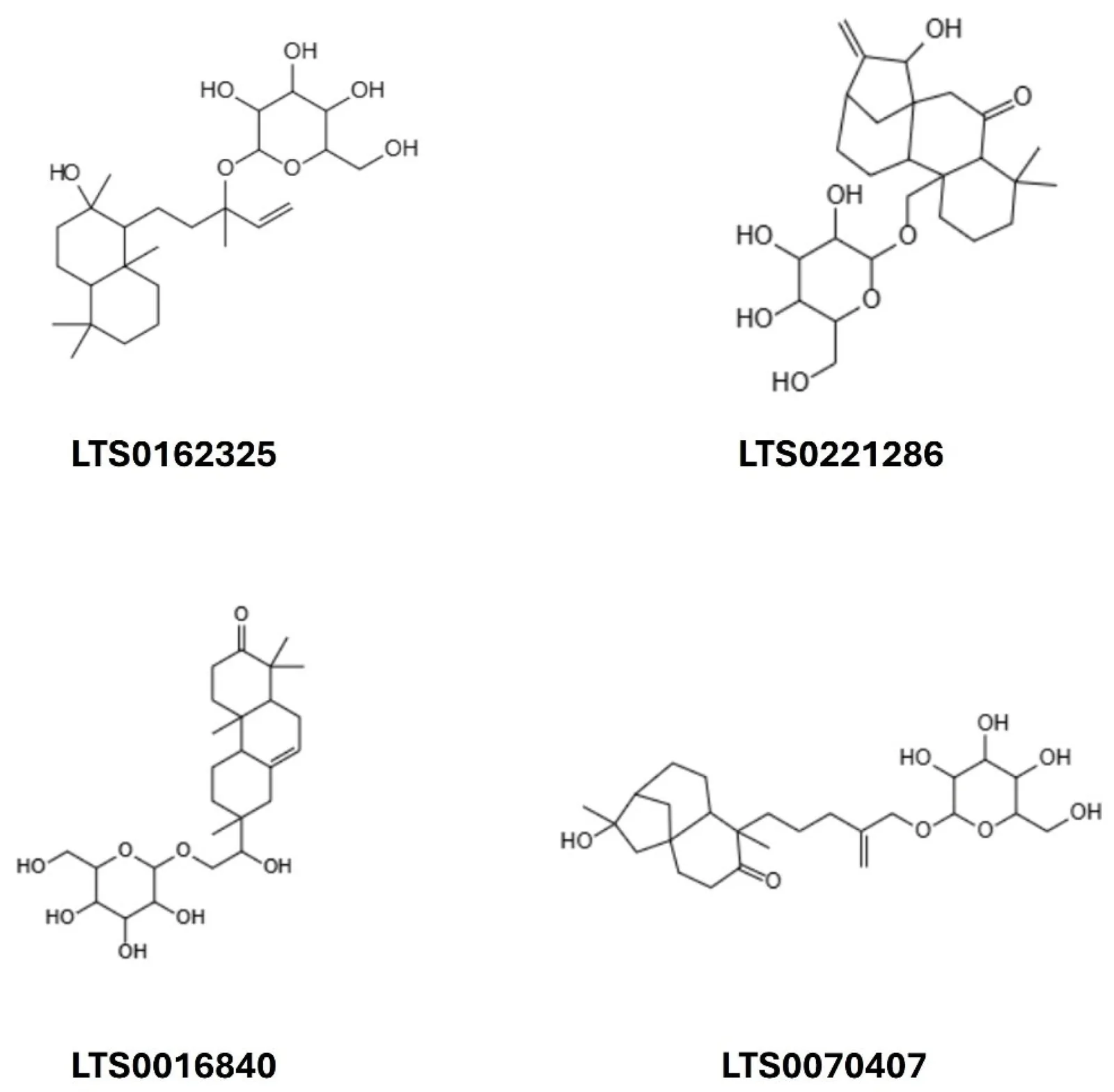
Chemical structure of lead compounds.

Among the identified hits, LTS0070407, LTS0091693, and LTS0140410 were the most promising candidates. These compounds belong to distinct natural product classes, including diterpenoid and coumarin glycosides [71,72,73], highlighting the structural diversity of potential caspase-1 inhibitors (Figure 12). The general information and predicted pKi values related to these compounds are shown in Table 4 and Table S3.

LTS0162325 is a class of Labdane diterpenoids that are synthesized by the *Sticherus quadripartitus* species [73]. Labdanes, as a family of bicyclic diterpenoid groups, indicate a broad range of biological effects including antibacterial, antiviral, anti-inflammatory, cytotoxic, and anti-tumor effects [74,75]. Therefore, they have potential to be used in pharmacological research [76]. Structurally, LTS0162325 possesses multiple hydroxyl functionalities distributed across a rigid diterpenoid scaffold, enabling the formation of an extensive hydrogen-bonding network with catalytic residues of caspase-1. From the QSAR perspective, the oxygen-rich molecular framework is compatible with descriptors that positively contributed to inhibitory activity, particularly F05[O–O], WiA_Coulomb, and RDF110s, reflecting favorable oxygen atom distribution, electrostatic interactions, and three-dimensional spatial organization. Furthermore, its extended diterpenoid skeleton is consistent with the positive contribution of the DISPm descriptor, suggesting that an increased spatial distribution of functional groups facilitates productive interactions within the caspase-1 binding pocket.

LTS0221286 is a kaurane/phyllocladane-type diterpenoid isolated from the liverwort *Jungermannia infusca*, which is recognized as a rich source of structurally diverse diterpenoids [77]. These types of diterpenoids indicate significant anti-inflammatory effects by lowering nitric oxide production, blocking inflammatory cytokines, and inhibiting the NF-κB signaling pathway [78,79]. This compound contains an oxygenated diterpenoid framework capable of establishing persistent hydrogen-bonding interactions with residues surrounding the catalytic pocket of caspase-1. Its structural architecture agrees well with several descriptors showing positive contributions in the developed QSAR model, including WiA_Coulomb, F05[O–O], and DLS_02, indicating that the spatial distribution of oxygen atoms, favorable electrostatic properties, and drug-like molecular characteristics contribute to its predicted activity. The rigid diterpenoid core combined with appropriately positioned oxygen-containing functional groups likely enhances complementarity with the active site while maintaining favorable molecular topology required for stable binding.

LTS0016840 is a class of Pimarane and Isopimarane diterpenoids that are synthesized by the *Palafoxia arida* species [80]. Pimarane and Isopimarane diterpenoids are plant natural compounds indicating strong anti-inflammatory activity by immune pathways such as NF-κB and lowering nitric oxide (NO) production [81,82,83]. The compact diterpenoid framework of LTS0016840, together with multiple oxygen-containing substituents, appears to provide a favorable balance between molecular rigidity and hydrogen-bonding capability. Accordingly, its structural features are consistent with the positive QSAR descriptors WiA_Coulomb, F05[O–O], and DISPm, suggesting that electrostatic complementarity, oxygen atom distribution, and three-dimensional molecular geometry contribute to the predicted inhibitory activity. The absence of excessive aromatic functionality may additionally minimize the influence of negatively contributing descriptors such as nArCO, further supporting its favorable QSAR profile.

LTS0070407, known as Pierisformoside G, a diterpene glycoside, was isolated from the leaves of *Pieris formosa* (family Ericaceae), a poisonous plant widely distributed in the mountainous and valley regions of southern and southwestern China. This compound represents a novel 5,6-seco-ring-A kaurane diterpenoid glucoside, a structural type that differs from previously reported grayanane and leucothane diterpenoids. Structurally, pierisformoside G possesses a glucose moiety attached to a kaurane-derived diterpenoid skeleton and contains characteristic functional groups, including hydroxyl, carbonyl, and double bonds. Plants of the genus Pieris produce a variety of biologically active diterpenoids, many of which exhibit toxic or defensive properties. Traditionally, the fresh leaf extract of *P. formosa* has been used as an insecticide and as a topical preparation for the treatment of skin diseases, such as tinea and scabies. Therefore, diterpenoid glycosides, such as pierisformoside G, are important natural products that contribute to the chemical diversity and biological activity of Pieris species [71]. Numerous species within these toxic genera are utilized in Chinese herbal medicine for the treatment of inflammation, asthma, and coughs. Contemporary research has revealed that the harmful components of these toxic genera are primarily tetracyclic diterpenes, which negatively impact the digestive, cardiovascular, and nervous systems. Despite their toxicity, various species of these genera possess medicinal properties, prompting extensive research that has uncovered a wide array of compounds [83]. Moreover, LTS0070407 is compatible with descriptors which have positive contributions in the QSAR model. Its oxygen-rich and flexible structure likely contributes to favorable electrostatic interactions and hydrogen bonding, consistent with the positive contributions of descriptors such as F05[O–O] and WiA_Coulomb.

Overall, the identification of diterpenoids highlights the potential of natural products as valuable sources of structurally diverse and biologically active compounds. These compounds should be considered as prioritized candidates for further experimental validation rather than as confirmed inhibitors.

Since caspase-1 plays a central role in inflammatory signaling through the maturation of pro-inflammatory cytokines, such as IL-1β and IL-18, the identification of natural diterpenoids as potential inhibitors may provide valuable insights for the development of novel anti-inflammatory therapeutics. Despite the promising computational results, the identified hits should be considered prioritized candidates for future in vitro caspase-1 inhibition and selectivity studies rather than confirmed inhibitors. Although the present study specifically focused on caspase-1, future studies may evaluate whether these compounds can be TNF-α inhibitors. Such analyses could clarify their potential as broader multitarget anti-inflammatory candidates.

## 4. Conclusions

In this study, an integrated computational workflow incorporating QSAR modeling alongside molecular docking, MM/GBSA analysis, filtering strategies, and molecular dynamics simulations was successfully applied to identify novel caspase-1 inhibitors from natural products. The PLS-based QSAR model was derived using the dataset of 185 compounds and 5799 descriptors for each, with high robustness and accuracy by statistical metrics (R^2^ = 0.870, Q^2^ = 0.819). Starting from a large dataset of 276,518 compounds from the LOTUS database, sequential filtering steps including Lipinski’s rule of five, QSAR-based virtual screening, molecular docking and MM/GBSA analysis, PAINS filtering, structural refinement, and ChemFH evaluation progressively reduced the dataset, ultimately yielding 14 top candidate compounds selected for molecular dynamics simulations.

Among these, four compounds (LTS0162325, LTS0221286, LTS0016840, and LTS0070407) exhibited the most stable binding behavior, maintaining persistent interactions with the key catalytic and substrate-binding residues of caspase-1. Overall, this hierarchical screening strategy efficiently reduced the chemical space while identifying promising lead candidates for further in vitro and in vivo experimental validation.

Descriptor-level interpretation further demonstrated that electrostatic interactions, spatial distribution of functional groups, and molecular flexibility play critical roles in determining the inhibitory activity. The identification of structurally diverse scaffolds, particularly diterpenoid and coumarin glycosides, emphasizes the potential of natural products as valuable sources of anti-inflammatory agents that target caspase-1.

## Figures and Tables

**Table 1:** Statistical parameters of the PLS-QSAR model and Y-randomization test.

	PLS Model		Y-Randomization		
Score	Training	Test	Min.	Mean	Max.
R^2^	0.870	0.838	0.020	0.063	0.177
Adjusted R^2^	0.850	0.741	0.000	0.002	0.051
Q^2^ LOO	0.819	-	0.000	0.001	0.036
Q^2^ 5-fold	0.814	-	0.000	0.002	0.057
RMSE	0.462	0.535	1.162	1.240	1.269
MAE	0.368	0.449	0.936	1.028	1.069

R^2^: coefficient of determination; Q^2^_LOO_: leave-one-out cross-validated coefficient; Q^2^_5-fold_: 5-fold cross-validated coefficient; RMSE: root mean square error; MAE: mean absolute error.

**Table 3:** Docking scores (LF VScore), MM/GBSA binding energies, and ChemFH filtering results of the top 14 compounds selected for molecular dynamics simulations.

LOTUS ID	LF VSscore (kcal/mol)	MM/GBSA Score (kcal/mol)	ChemFH Criteria	MM/GBSA Dynamics (kcal/mol)
LTS0274950	−12.617	−62.04	Pass	−33.01
LTS0080339	−14.929	−61.74	Pass	−25.06
LTS0091693	−12.997	−58.96	Pass	−30.76
LTS0070407	−14.05	−58.66	Pass	−34.33
LTS0016840	−13.301	−57.81	Pass	−35.05
LTS0140410	−13.196	−55.74	Pass	−32.36
LTS0050358	−13.606	−53.56	Pass	−14.37
LTS0170777	−13.686	−53.11	Pass	−15.5
LTS0059426	−13.147	−52.52	Pass	−22.1
LTS0162325	−13.181	−51.97	Pass	−39.81
LTS0221286	−13.565	−51.89	Pass	−36.23
LTS0115315	−13.388	−51.31	Pass	−30.34
LTS0199816	−13.301	−50.79	Pass	−28.34
LTS0029227	−13.371	−50.62	Pass	−31.53
Native ligand	−11.724	−58.42		−46.2

LF VScore: LeadFinder docking score; MM/GBSA: Molecular Mechanics/Generalized Born Surface Area binding free energy estimation; ChemFH: Chemical Frequent Hitter.

**Table 4:** Top natural product hits from the LOTUS database identified by the QSAR model and their predicted pKi values.

No	Species	LOTUS ID	Predicted pKi
1	*Sticherus quadripartitus*	LTS0162325	9.93
2	*Palafoxia arida*	LTS0016840	10.82
3	*Jungermannia infusca*	LTS0221286	10.03
4	*Pieris formosa*	LTS0070407	9.88

## Data Availability

The datasets supporting the conclusions of this article are included within the article.

## Supplementary Materials

The following supporting information can be downloaded at https://www.mdpi.com/article/10.3390/molecules31162894/s1. Figure S1: Histogram shows the distribution of pKi values for the caspase-1 inhibitors in the dataset. The x-axis represents the pKi values (inhibitory activity), while the y-axis indicates the frequency of compounds within each activity interval. Figure S2: Molecular dynamics analysis of the apo and co-crystallized ligand-bound forms of caspase-1 (PDB ID: 6PZP). (A) RMSD profile calculated for the displayed heavy atoms of the apo caspase-1 system over the 300 ns MD simulation. (B) RMSF profile of apo caspase-1. (C) RMSD profile calculated for the displayed heavy atoms of the co-crystallized ligand-bound caspase-1 complex over the 300 ns MD simulation. (D) RMSF profile of the co-crystallized ligand-bound caspase-1 complex. Table S1: SMILES, experimental and predicted pKi values, and residuals of the caspase-1 inhibitors used for the development of the QSAR model. Table S2: The values of selected descriptors for all compounds. Table S3: Top 14 natural product hits from the LOTUS database identified by the QSAR model and their predicted pKi values.
