# Supplementary material for: A Comprehensive Study Utilizing QSAR, Virtual Screening, Molecular Docking, Molecular Dynamics, and MM/GBSA Analyses Reveals Natural Diterpenoids as Promising Caspase-1 Inhibitors
Source: Molecules. 2026 Aug 19;31(16):2894. doi: 10.3390/molecules31162894 (PMC13515187; doi:10.3390/molecules31162894)
Supplement: Supplementary file 1 [file molecules-31-02894-s001.zip › molecules-4489849-supplementary.pdf]

A comprehensive study utilizing QSAR, virtual screening, molecular docking, molecular dynamics, and MM/GBSA analyses reveals natural diterpenoids as promising caspase-1 inhibitors

Yusuf Şeflekçi[1,2]*, Alper Yılmaz[2], Abdulilah Ece[3]

[1]Department of Molecular Biology and Genetics, Faculty of Arts and Sciences, Yıldız Technical University, İstanbul, Türkiye

[2]Department of Molecular Biology and Genetics, Faculty of Engineering and Natural Sciences, Biruni University, İstanbul, 34015, Türkiye

[3]Department of Medical Biochemistry, Faculty of Medicine, Biruni University, İstanbul, 34015, Türkiye

Corresponding Author:

Yusuf Şeflekçi

Department of Molecular Biology and Genetics, Faculty of Engineering and Natural Sciences, Biruni University, , İstanbul, 34015, Türkiye

E-mail: yseflekci@biruni.edu.tr

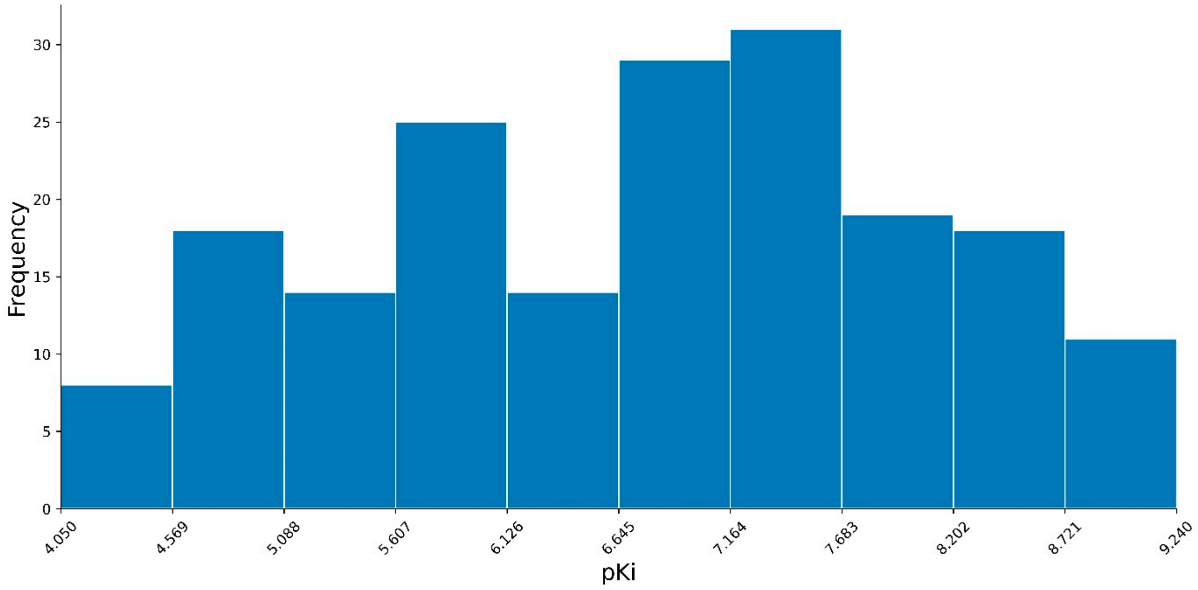

Figure S1. Histogram shows the distribution of pKi values for the caspase-1 inhibitors in the dataset. The x-axis represents the pKi values (inhibitory activity), while the y-axis indicates the frequency of compounds within each activity interval.

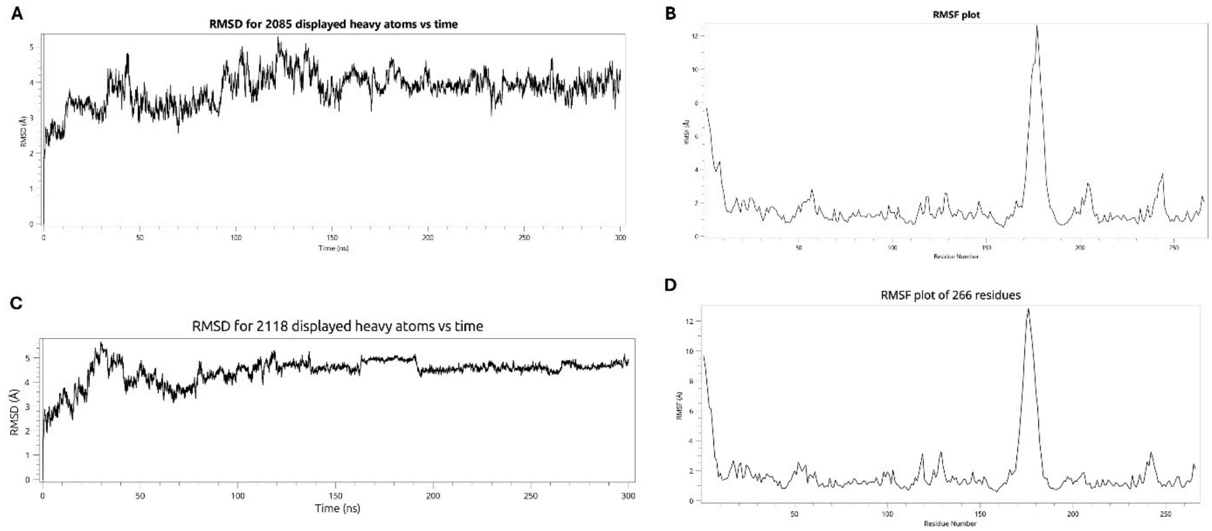

Figure S2. Molecular dynamics analysis of the apo and co-crystallized ligand-bound forms of caspase-1 (PDB ID: 6PZP). **(A)** RMSD profile calculated for the displayed heavy atoms of the apo caspase-1 system over the 300 ns MD simulation. **(B)** RMSF profile of apo caspase-1. **(C)** RMSD profile calculated for the displayed heavy atoms of the co-crystallized ligand-bound caspase-1 complex over the 300 ns MD simulation. **(D)** RMSF profile of the co-crystallized ligand-bound caspase-1 complex.

Table S1. SMILES format, calculated experimental pKi, predicted pKi and residuals of all compounds

No.	SMILES	Experimental pKi (nM)	Predicted pKi	Residual
0	[O-]C(=O)CC(NC(=O)OC\C=C)C(=O)COC(=O)C=Cc1ccccc1	4.05	4.771	-0.721
1	[O-]C(=O)CC(NC(=O)OCc1ccccc1)C(=O)CON1CCCC1=O	4.06	4.202	-0.142
1	[O-]C(=O)CC(NC(=O)OCc1ccccc1)C(=O)CONC(=O)Cc1cccc2ccccc12	4.08	4.662	-0.582
1	[O-]C(=O)CC(NC(=O)OCc1ccccc1)C(=O)CONC(=O)Cc1ccccc1	4.18	4.36	-0.18
1	[O-]C(=O)CC(NC(=O)OCc1ccccc1)C(=O)CNS(=O)(=O)c1ccccc1	4.43	4.58	-0.15
1	[O-]C(=O)CC(NC(=O)OCc1ccccc1)C(=O)CNS(=O)(=O)CCCc1ccccc1	4.46	4.675	-0.215
0	[O-]C(=O)CC(NC(=O)OCc1ccccc1)C(=O)CNS(=O)(=O)CCC1CCCCC1	4.48	4.549	-0.069
1	[O-]C(=O)CC(NC(=O)OCc1ccccc1)C(=O)CONC(=O)CCc1ccccc1	4.56	4.514	0.046
1	[O-]C(=O)CC(NC(=O)OCc1ccccc1)C(=O)COCCCc1ccccc1	4.62	4.59	0.03
1	[O-]C(=O)CC(NC(=O)OC/C=C)C(=O)COC(=O)CCOc1ccccc1	4.64	4.989	-0.349
0	[O-]C(=O)CC(NC(=O)OCc1ccccc1)C(=O)CNS(=O)(=O)c1cccc2ccccc12	4.66	4.924	-0.264
1	[O-]C(=O)CC(NC(=O)OCc1ccccc1)C(=O)CONC(=O)c1ccccc1	4.68	4.741	-0.061
1	[O-]C(=O)CC(NC(=O)OC\C=C)C(=O)COC(=O)CCc1ccccc1	4.7	5.368	-0.668
1	[O-]C(=O)CC(NC(=O)OCc1ccccc1)C(=O)CON1C2CCCCC2CC1=O	4.76	5.249	-0.489
0	[O-]C(=O)CC(NC(=O)OCc1ccccc1)C(=O)CNS(=O)(=O)CCc1cccc2ccccc12	4.8	5.092	-0.292
1	[O-]C(=O)CC(NC(=O)OCc1ccccc1)C(=O)COC(=O)CCCc1ccccc1	4.8	4.997	-0.197
0	[O-]C(=O)CC(NC(=O)OCc1ccccc1)C(=O)CON1Cc2ccccc2C1=O	4.85	5.27	-0.42
1	CC(C)C(NC(=O)OCc1ccccc1)C(=O)NC(C)C(=O)NC(CC([O-])=O)C(=O)c1cocn1	4.85	5.097	-0.247
1	CC(C)C(NC(=O)OCc1ccccc1)C(=O)NC(C)C(=O)NC(CC([O-])=O)C(=O)c1ncco1	4.89	5.042	-0.152
1	[O-]C(=O)CC(NC(=O)OCc1ccccc1)C(=O)CNS(=O)(=O)CCc1ccccc1	4.96	4.875	0.085
1	CC(C)C(NC(=O)OCc1ccccc1)C(=O)NC(C)C(=O)NC(CC([O-])=O)C(=O)c1nccs1	5	4.696	0.304
0	[O-]C(=O)CC(NC(=O)OC/C=C)C(=O)COc1cccc2ccccc12	5.02	4.934	0.086
1	[O-]C(=O)CC(NC(=O)OCc1ccccc1)C(=O)CON1C2CCCCC2CC1=O	5.04	5.199	-0.159
1	CC(C)C(NC(=O)OCc1ccccc1)C(=O)NC(C)C(=O)NC(CC([O-])=O)C(=O)c1ncc[nH]1	5.06	4.911	0.149
1	Cc1cccc2cccc(c12)CC(=O)OCC(=O)C(CC([O-])=O)NC(=O)OCc1ccccc1	5.07	5.616	-0.546
0	[O-]C(=O)CC(NC(=O)OCc1ccccc1)C(=O)COC(=O)Cc1ccccc1	5.08	5.385	-0.305
0	[O-]C(=O)CC(NC(=O)OCc1ccccc1)C(=O)CON1C(=O)Cc2ccccc12	5.17	4.551	0.619
1	CC(C)C(NC(=O)OCc1ccccc1)C(=O)NC(C)C(=O)NC(CC([O-])=O)C(=O)c1nc(c[nH]1)-c1ccccc1	5.2	5.248	-0.048
0	[O-]C(=O)CC(NC(=O)OCc1ccccc1)C(=O)COC(=O)CCc1ccc2ccccc2c1	5.22	6.03	-0.81
1	[O-]C(=O)CC(NC(=O)OCc1ccccc1)C(=O)COC(=O)CNc1ccccc1	5.25	4.98	0.27

1	[O-]C(=O)CC(NC(=O)OCc1ccccc1)C(=O)COC(=O)CCc1ccccc1	5.35	5.689	-0.339
0	[O-]C(=O)CC(NC(=O)OC/C=C)C(=O)C[NH2+]CCc1ccccc1	5.36	5.108	0.252
1	Cc1ccc2ccccc2c1CC(=O)OCC(=O)C(CC([O-])=O)NC(=O)OCc1ccccc1	5.36	5.837	-0.477
1	[O-]C(=O)CC(NC(=O)OC/C=C)C(=O)COc1ccc2ccccc2c1	5.4	5.026	0.374
0	NC(=O)c1ccccc1OCC(=O)C(CC([O-])=O)NC(=O)OC\C=C	5.42	5.002	0.418
1	[O-]C(=O)CC(NC(=O)OC\C=C)C(=O)COC(=O)CCC1CCCCC1	5.46	4.862	0.598
0	[O-]C(=O)CC(NC(=O)CCCCNC(=O)c1ccc(cc1)Nc1cnc2ccccc2n1)C=O	5.55	6.415	-0.865
1	[O-]C(=O)CC(NC(=O)CCCCCNC(=O)c1ccc(cc1)Nc1cnc2ccccc2n1)C=O	5.58	6.201	-0.621
0	[O-]C(=O)CC(NC(=O)OCc1ccccc1)C(=O)COC(=O)Cc1cccc2scnc12	5.58	5.309	0.271
1	COC(=O)c1cc2ccccc2cc1OCC(=O)C(CC([O-])=O)NC(=O)OC\C=C	5.58	5.68	-0.1
1	[O-]C(=O)CC(NC(=O)CCCCNC(=O)c1ccc(cc1)Oc1cnc2ccccc2n1)C=O	5.62	6.616	-0.996
0	NC(=O)c1ccc(cc1OCC(=O)C(CC([O-])=O)NC(=O)OC\C=C)-c1ccccc1	5.7	5.413	0.287
1	Cc1cccc2c(cccc12)CC(=O)OCC(=O)C(CC([O-])=O)NC(=O)OCc1ccccc1	5.7	5.523	0.177
1	[O-]C(=O)CC(NC(=O)C1CCC(CC1)CNC(=O)c1ccc(cc1)Nc1cnc2ccccc2n1)C=O	5.71	6.603	-0.893
0	CC(C)C(NC(=O)c1ccc2ccccc2c1)C(=O)NC(C)C(=O)NC(C[NH+](C)Cc1ccccc1)CC([O-])=O	5.72	6.247	-0.527
1	CC(C)C(NC(=O)c1ccc2ccccc2c1)C(=O)NC(C)C(=O)NC(C[NH2+]Cc1ccc(cc1)C(N)=O)CC([O-])=O	5.74	6.316	-0.576
1	[O-]C(=O)CC(NC(=O)OCc1ccccc1)C(=O)COC(=O)COc1ccccc1	5.77	5.464	0.306
1	[O-]C(=O)CC(NC(=O)OCc1ccccc1)C(=O)CON1C(=O)C2C3OC(C=C3)C2C1=O	5.82	6.029	-0.209
1	CC(C)C(NC(=O)c1ccc2ccccc2c1)C(=O)NC(C)C(=O)NC(C[NH2+]Cc1ccc(cc1)O)CC([O-])=O	5.86	6.046	-0.186
0	[O-]C(=O)CC(NC(=O)OCc1ccccc1)C(=O)COC(=O)Cc1ccc2ccccc2c1	5.89	6.058	-0.168
1	CC(C)C(NC(=O)OCc1ccccc1)C(=O)NCC(=O)NC(CC([O-])=O)C([O-])=O	5.89	6.662	-0.772
1	Cc1ccc(c2ccccc12)CC(=O)OCC(=O)C(CC([O-])=O)NC(=O)OCc1ccccc1	5.89	5.55	0.34
0	[O-]C(=O)CC(NC(=O)CN1C(=CC=C(NC(=O)CCc2ccccc2)C1=O)CCc1ccccc1)C=O	5.92	6.178	-0.258
1	OCc1cc2ccccc2cc1OCC(=O)C(CC([O-])=O)NC(=O)OC/C=C	5.92	5.65	0.27
1	[O-]C(=O)CC(NC(=O)OCc1ccccc1)C(=O)COC(=O)Cc1csc2ccccc12	5.92	5.535	0.385
1	CC(C)C(NC(=O)c1ccc2ccccc2c1)C(=O)NC(C)C(=O)NC(C[NH2+]Cc1ccccc1F)CC([O-])=O	5.92	6.225	-0.305
1	[O-]C(=O)CC(NC(=O)OCc1ccccc1)C(=O)COC(=O)CC(c1ccccc1)c1ccccn1	5.96	5.84	0.12
0	CCCCC1=CC=C(NC(=O)CCc2ccccc2)C(=O)N1CC(=O)NC(CC([O-])=O)C=O	6	6.107	-0.107
1	CC(C(=O)OCC(=O)C(CC([O-])=O)NC(=O)OCc1ccccc1)c1cccc2ccccc12	6	5.802	0.198
1	[O-]C(=O)CC(NC(=O)OCc1ccccc1)C(=O)COC(=O)COc1cccc2ccccc12	6	6.285	-0.285
1	[O-]C(=O)CC(NC(=O)c1cnc(cn1)CNC(=O)c1ccc(cc1)Nc1cnc2ccccc2n1)C=O	6	6.324	-0.324
1	CC(N1C(=CC=C(NC(=O)CCc2ccccc2)C1=O)Cc1ccccc1)C(=O)NC(CC([O-])=O)C=O	6.01	6.198	-0.188
1	CC(C)C(NC(=O)c1ccc2ccccc2c1)C(=O)NC(C)C(=O)NC(C[NH2+]C1CCCCC1)CC([O-])=O	6.03	6.421	-0.391
1	[O-]C(=O)CC(NC(=O)CN1C(=O)C(=CC=C1c1ccccc1)NC(=O)CCc1ccccc1)C=O	6.05	6.017	0.033
0	[O-]C(=O)CC(NC(=O)OCc1ccccc1)C(=O)COC(=O)Cc1coc2ccccc12	6.1	6.446	-0.346

0	CC(C)C(NC(=O)c1ccc2ccccc2c1)C(=O)NC(C)C(=O)NC(C[NH2+]Cc1ccccc1)CC([O-])=O	6.22	6.331	-0.111
1	[O-]C(=O)CC(NC(=O)OCc1ccccc1)C(=O)COC(=O)Cc1c[nH]c2ccccc12	6.22	5.705	0.515
1	CC1=CC=C(NC(=O)OCc2ccccc2)C(=O)N1CC(=O)NC(CC([O-])=O)C(=O)COc1ccccc1	6.23	5.972	0.258
1	CC1=CC=C(NC(=O)c2ccccc2)C(=O)N1CC(=O)NC(CC([O-])=O)C(=O)COc1ccccc1	6.28	6.552	-0.272
0	[O-]C(=O)CC(NC(=O)OC\C=C)C(=O)COc1cc2ccccc2cc1C(=O)Nc1ccccc1	6.3	5.427	0.873
1	[O-]C(=O)CC(NC(=O)OCc1ccccc1)C(=O)COC(=O)Cc1cccc2ccccc12	6.3	5.983	0.317
0	CCC(N1C=CC=C(NC(=O)c2ccc3ccccc3c2)C1=O)C(=O)NC(CC([O-])=O)C(=O)C[NH+]1CCNCC1	6.39	7.001	-0.611
1	CCC(N1C=CC=C(NC(=O)c2ccc3ccccc3c2)C1=O)C(=O)NC(CC([O-])=O)C(=O)CN1C=CC(=O)C=C1	6.4	7.18	-0.78
1	NC(=O)c1cc2ccccc2cc1OCC(=O)C(CC([O-])=O)NC(=O)OC/C=C	6.5	5.724	0.776
0	Cc1cc(ccc1Nc1cnc2ccccc2n1)C(=O)NCCCCCC(=O)NC(CC([O-])=O)C=O	6.5	6.787	-0.287
1	C1CCN(C1)c1cc(nc(n1)N1CCCC1)N1CC[NH+](CC1)Cc1ccncc1	6.52	6.813	-0.293
1	CCC(N1C=CC=C(NC(=O)c2ccc3ccccc3c2)C1=O)C(=O)NC(CC([O-])=O)C(=O)C[NH+](C)C(Cc1ccccc1)C(N)=O	6.59	7.086	-0.496
1	O=N(=O)c1ccccc1C[NH+]1CCN(CC1)c1cc(nc(n1)N1CCCC1)N1CCCC1	6.64	7.244	-0.604
1	CC(C)C(NC(=O)c1ccc2ccccc2c1)C(=O)NC(C)C(=O)NC(C[NH2+]Cc1ccc2c(c1)OCO2)CC([O-])=O	6.68	6.928	-0.248
1	C1CCN(C1)c1cc(nc(n1)N1CCCC1)N1CC[NH+](CC1)Cc1ccccc1	6.7	6.721	-0.021
1	[O-]C(=O)CC(NC(=O)CCCCCNC(=O)c1ccc(cc1)Nc1cnc2ccccc2n1)C=O	6.72	6.581	0.139
1	FC(F)(F)c1ccc(cc1)C[NH+]1CCN(CC1)c1cc(nc(n1)N1CCCC1)N1CCCC1	6.74	6.526	0.214
1	CC(C)C(NC(=O)C(Cc1ccc(cc1)O)NC(=O)OCc1ccccc1)C(=O)NC(C)C(=O)NC(CC([O-])=O)C=O	6.75	7.285	-0.535
0	CC1=CC=C(NC(=O)c2ccc3ccccc3c2)C(=O)N1CC(=O)NC(CC([O-])=O)C(=O)COc1ccccc1	6.8	7.126	-0.326
1	CC(N1C=CC=C(NC(=O)CCc2ccccc2)C1=O)C(=O)NC(CC([O-])=O)C=O	6.82	6.913	-0.093
0	[O-]C(=O)CC(NC(=O)CN1C(=CC=C(NC(=O)CCc2ccccc2)C1=O)Cc1ccccc1)C=O	6.82	6.419	0.401
1	CC(C)C(NC(=O)OCc1ccccc1)C(=O)NC(C)C(=O)NC(CC([O-])=O)C(=O)CCCCCc1ccccc1	6.87	6.943	-0.073
1	CC(C)C(NC(=O)OCc1ccccc1)C(=O)NC(C)C(=O)NC(CC([O-])=O)C(=O)CCCCCc1ccccc1	6.87	6.797	0.073
1	O=N(=O)c1ccc(cc1)C[NH+]1CCN(CC1)c1cc(nc(n1)N1CCCC1)N1CCCC1	6.89	6.673	0.217
1	CC(C)C(NC(=O)c1ccc2ccccc2c1)C(=O)NC(C)C(=O)NC(C[NH+](C)C1CCCCC1)CC([O-])=O	6.89	6.326	0.564
1	CC(C)C(NC(=O)CCc1ccccc1)C(=O)NC(C)C(=O)NC(CC([O-])=O)C(=O)COC(=O)NCc1ccccc1	6.92	7.508	-0.588
0	[O-]C(=O)CC(NC(=O)c1csc(c1)CNC(=O)c1ccc(cc1)Nc1cnc2ccccc2n1)C=O	6.92	6.792	0.128
1	[O-]C(=O)CC(NC(=O)Cc1ccc(s1)CNC(=O)c1ccc(cc1)Nc1cnc2ccccc2n1)C=O	6.92	6.78	0.14
1	CCC(N1C=CC=C(NC(=O)c2ccc3ccccc3c2)C1=O)C(=O)NC(CC([O-])=O)C(=O)CNC(C)=O	6.93	7.595	-0.665
1	CC(C)C(CC(=O)NCCCc1ccccc1)C(=O)NC(CC([O-])=O)C=O	6.94	6.832	0.108
1	CC(C)C(NC(=O)CCc1ccccc1)C(=O)NC(C)C(=O)NC(CC([O-])=O)C(=O)CS(=O)(=O)CCCc1ccccc1	6.95	7.909	-0.959
0	CC(C)C(NC(=O)CCc1ccccc1)C(=O)NC(C)C(=O)NC(CC([O-])=O)C(=O)CCCCc1ccccc1	7	7.935	-0.935
1	[O-]C(=O)CC(NC(=O)c1ccc(cc1)CNC(=O)c1ccc(cc1)Nc1cnc2ccccc2n1)C=O	7	6.78	0.22
1	[O-]C(=O)CC(NC(=O)C(CCCCNC(=O)c1ccc(cc1)Nc1cnccn1)c1cccs1)C=O	7	7.27	-0.27
1	CC1=CC=C(NC(=O)c2cccc3ccccc23)C(=O)N1CC(=O)NC(CC([O-])=O)C(=O)COc1ccccc1	7.02	7.102	-0.082

1	OC1OC(=O)CC1NC(=O)CN1C(=O)C(CN(C(=O)CCc2ccccc2)c2ccccc12)NC(=O)c1ccccc1	7.05	8.047	-0.997
1	CC(C)C(NC(C)=O)C(=O)NC(CC([O-])=O)C(=O)COC(=O)Cc1c(cccc1Cl)Cl	7.09	6.993	0.097
0	CCCC(=O)NCC(=O)C(CC([O-])=O)NC(=O)C(CC)N1C=CC=C(NC(=O)c2ccc3ccccc3c2)C1=O	7.09	7.823	-0.733
1	CCC(N1C=CC=C(NC(=O)c2ccc3ccccc3c2)C1=O)C(=O)NC(CC([O-])=O)C(=O)C[NH2+]C(Cc1ccccc1)C(N)=O	7.11	6.687	0.423
1	COc1cccc(c1)C[NH+]1CCN(CC1)c1cc(nc(n1)N1CCCC1)N1CCCC1	7.12	7.083	0.037
0	COc1ccc(cc1)CCCNC(=O)CC(C(C)C)C(=O)NC(CC([O-])=O)C=O	7.15	7.515	-0.365
1	CCCC1=CC=C(NC(=O)c2cccc3ccccc23)C(=O)N1CC(=O)NC(CC([O-])=O)C(=O)COc1ccccc1	7.17	7.107	0.063
1	[O-]C(=O)CC(NC(=O)CN1C(=O)C(=CN=C1c1ccccc1)NC(=O)c1cccc2ccccc12)C(=O)COc1ccccc1	7.18	7.199	-0.019
0	CCC(N1C=CC=C(NC(=O)c2ccc3ccccc3c2)C1=O)C(=O)NC(CC([O-])=O)C(=O)C[NH+]1CCN(C)CC1	7.2	7.25	-0.05
1	CC(C)C(NC(=O)CCc1ccccc1)C(=O)NC(C)C(=O)NC(CC([O-])=O)C(=O)C[NH+](CCc1ccccc1)Cc1ccccc1	7.22	7.879	-0.659
0	CCC(N1C=CC=C(NC(=O)c2ccc3ccccc3c2)C1=O)C(=O)NC(CC([O-])=O)C(=O)C[NH+](C)Cc1ccccc1	7.24	7.319	-0.079
1	CCC(N1C=CC=C(NC(=O)c2ccc3ccccc3c2)C1=O)C(=O)NC(CC([O-])=O)C(=O)CNC(=O)c1ccccc1	7.24	7.797	-0.557
1	CC(C)C(NC(=O)OCc1ccccc1)C(=O)NC(C)C(=O)NC(CC([O-])=O)C([O-])=O	7.26	7.16	0.1
1	CC(=O)NC(C(=O)NC1=CC=C(Cc2ccccc2)N(CC(=O)NC(CC([O-])=O)C=O)C1=O)c1ccc(cc1)O	7.27	8.121	-0.851
1	[O-]C(=O)CC(NC(=O)C(CCCCNC(=O)c1ccc(cc1)Nc1cncc(n1)Cl)c1cccs1)C=O	7.27	7.474	-0.204
0	CC12CC=C3C(CCC4=CC(=O)C=CC34C)C2CCC1C(=O)C[NH+]1CCN(CC1)c1cc(nc(n1)N1CCCC1)N1CCCC1	7.3	8.733	-1.433
1	CC12CC=C3C(CCC4=CC(=O)C=CC34C)C2CCC1C(=O)C[NH+]1CCN(CC1)c1cc(nc(n1)N1CCCC1)N1CCCC1	7.3	8.537	-1.237
1	COc1cc(cc(c1)OC)C[NH+]1CCN(CC1)c1cc(nc(n1)N1CCCC1)N1CCCC1	7.31	7.268	0.042
1	CC(C)C(NC(=O)c1ccc2ccccc2c1)C(=O)NC(C)C(=O)NC(C[NH2+]Cc1ccccc1O)CC([O-])=O	7.33	6.061	1.269
1	CCCCC1=NC=C(NC(=O)c2cccc3ccccc23)C(=O)N1CC(=O)NC(CC([O-])=O)C(=O)COc1ccccc1	7.34	7.353	-0.013
1	CCC(N1C=CC=C(NC(=O)c2ccc3ccccc3c2)C1=O)C(=O)NC(CC([O-])=O)C(=O)C[NH+]1CCOCC1	7.35	7.156	0.194
1	[O-]C(=O)CC(NC(=O)CN1C=CC=C(NC(=O)c2ccc3ccccc3c2)C1=O)C(=O)COc1ccccc1	7.35	6.858	0.492
1	[O-]C(=O)CC(NC(=O)C(CCCCNC(=O)c1ccc(cc1)Oc1cnc2ccccc2n1)c1cccs1)C=O	7.37	7.929	-0.559
0	CCC1=CC=C(NC(=O)c2cccc3ccccc23)C(=O)N1CC(=O)NC(CC([O-])=O)C(=O)COc1ccccc1	7.4	7.219	0.181
1	CCCCCCC1=CC=C(NC(=O)c2cccc3ccccc23)C(=O)N1CC(=O)NC(CC([O-])=O)C(=O)COc1ccccc1	7.4	7.484	-0.084
1	[O-]C(=O)CC(NC(=O)CN1C=CC=C(NC(=O)c2cccc3ccccc23)C1=O)C(=O)COc1ccccc1	7.41	6.952	0.458
1	CCC(N1C=CC=C(NC(=O)c2ccc3ccccc3c2)C1=O)C(=O)NC(CC([O-])=O)C(=O)C[NH+](C)CCCC(N)=O	7.43	7.278	0.152
1	CCCCCC(=O)NCC(=O)C(CC([O-])=O)NC(=O)C(CC)N1C=CC=C(NC(=O)c2ccc3ccccc3c2)C1=O	7.46	7.865	-0.405
0	CC(C)C(NC(=O)CCc1ccccc1)C(=O)NC(C)C(=O)NC(CC([O-])=O)C(=O)CS(=O)CCCc1ccccc1	7.57	8.14	-0.57
1	C1CCC(CC1)C[NH+]1CCN(CC1)c1cc(nc(n1)N1CCCC1)N1CCCC1	7.57	6.747	0.823
0	[O-]C(=O)CC(NC(=O)C1CCCN2N1C(=O)C(CCC2=O)NC(=O)OCc1ccccc1)C=O	7.6	7.027	0.573
1	CCC(N1C=CC=C(NC(=O)c2ccc3ccccc3c2)C1=O)C(=O)NC(CC([O-])=O)C(=O)C[NH2+]C(Cc1ccccc1)C(N)=O	7.6	6.87	0.73
1	[O-]C(=O)CC(NC(=O)CN1C(=O)C(=CC=C1c1ccccc1)NC(=O)c1cccc2ccccc12)C(=O)COc1ccccc1	7.6	7.258	0.342
1	CC(NC(=O)C(CCC([O-])=O)NC(=O)OCc1ccccc1)C(=O)NC(CC([O-])=O)C(=O)CNS(=O)(=O)CCc1ccccc1	7.62	7.568	0.052
0	CCC(N1C=CC=C(NC(=O)c2ccc3ccccc3c2)C1=O)C(=O)NC(CC([O-])=O)C(=O)C[NH+](C)C	7.64	7.055	0.585

1	CC(C)C(CC(=O)N1CCCC(C1)Oc1ccccc1)C(=O)NC(CC([O-])=O)C=O	7.67	8.652	-0.982
1	CC(C)C(N1C=CC=C(NC(=O)c2cccc3ccccc23)C1=O)C(=O)NC(CC([O-])=O)C(=O)COc1ccccc1	7.68	7.598	0.082
0	CC(C)C(CC(=O)NCCn1cnc2ccccc12)C(=O)NC(CC([O-])=O)C=O	7.7	8.476	-0.776
1	CC(C)C(CC(=O)NCCOc1ccccc1)C(=O)NC(CC([O-])=O)C=O	7.77	7.627	0.143
1	CC(C)C(CC(=O)NCCc1cccc2nc(ccc12)OCC[NH+](C)C)C(=O)NC(CC([O-])=O)C=O	7.78	7.364	0.416
0	CCCCC1=CC=C(NC(=O)c2cccc3ccccc23)C(=O)N1CC(=O)NC(CC([O-])=O)C(=O)COc1ccccc1	7.8	7.278	0.522
1	CCC(CCCCNC(=O)c1ccc(cc1)Nc1cnc2ccccc2n1)C(=O)NC(CC([O-])=O)C=O	7.8	7.097	0.703
1	C1CCN(C1)c1cc(nc(n1)N1CCCC1)N1CC[NH+](CC1)CCc1ccccc1	7.8	6.962	0.838
0	CC(C)C(NC(=O)OCc1ccccc1)C(=O)NC(C)C(=O)NC(CC([O-])=O)C([O-])=O	7.85	7.429	0.421
1	CC(N1C=CC=C(NC(=O)c2ccc3ccccc3c2)C1=O)C(=O)NC(CC([O-])=O)C(=O)COc1ccccc1	7.94	7.124	0.816
0	CCC(N1C=CC=C(NC(=O)c2ccc3ccccc3c2)C1=O)C(=O)NC(CC([O-])=O)C(=O)C[NH+]1CCC(CC1)C(N)=O	7.95	7.173	0.777
1	CC(C)C(NC(=O)CCc1ccccc1)C(=O)NC(C)C(=O)NC(CC([O-])=O)C(=O)CSCCCc1ccccc1	7.96	8.008	-0.048
0	CC(C)C(NC(=O)C(Cc1ccc(cc1)O)NC(C)=O)C(=O)NC(C)C(=O)NC(CC([O-])=O)C(=O)CCCCCc1ccccc1	8	7.796	0.204
1	[O-]C(=O)CC(NC(=O)CN1C(=CC=C(NC(=O)c2cccc3ccccc23)C1=O)Cc1ccccc1)C(=O)COc1ccccc1	8.03	7.446	0.584
1	CC(C)C(CC(=O)N(CCOc1ccccc1)CCC([O-])=O)C(=O)NC(CC([O-])=O)C=O	8.05	7.639	0.411
0	CC(C)C(NC(=O)C(Cc1ccc(cc1)O)NC(C)=O)C(=O)NC(C)C(=O)NC(CC([O-])=O)C([O-])=O	8.1	8.804	-0.704
1	CCOC(=O)C1CC[NH+](CC1)CC(=O)C(CC([O-])=O)NC(=O)C(CC)N1C=CC=C(NC(=O)c2ccc3ccccc3c2)C1=O	8.1	7.649	0.451
1	CC(C)C(CC(=O)NCCc1c[nH]c2ccc(cc12)F)C(=O)NC(CC([O-])=O)C=O	8.14	8.669	-0.529
1	COCC(N1C=CC=C(NC(=O)c2ccc3ccccc3c2)C1=O)C(=O)NC(CC([O-])=O)C(=O)COc1ccccc1	8.15	7.784	0.366
1	CC(N1C=CC=C(NC(=O)c2cccc3ccccc23)C1=O)C(=O)NC(CC([O-])=O)C(=O)COc1ccccc1	8.15	7.354	0.796
1	COC(=O)CC1CCCC2CC(=O)N(OCC(=O)C(CC([O-])=O)NC(=O)C(C)NC(=O)C(NC(=O)OCc3ccccc3)C(C)C)C12	8.15	8.466	-0.316
1	CC(C)C(NC(C)=O)C(=O)NC(C)C(=O)NC(CC([O-])=O)C([O-])=O	8.28	8.384	-0.104
0	CCCCC(N1C=CC=C(NC(=O)c2cccc3ccccc23)C1=O)C(=O)NC(CC([O-])=O)C(=O)COc1ccccc1	8.3	7.457	0.843
1	CC(C)C(CC(=O)NCCc1c[nH]c2ccccc12)C(=O)NC(CC([O-])=O)C=O	8.3	8.149	0.151
1	[O-]C(=O)CC(NC(=O)C1CCCN2N1C(=O)C(CCC2=O)NC(=O)c1ccccc1)C=O	8.3	8.617	-0.317
1	CC(C)C(CC(=O)NCCn1cnc2cc(ccc12)C)C(=O)NC(CC([O-])=O)C=O	8.32	8.123	0.197
1	CC(C)C(NC(=O)CCc1ccccc1)C(=O)NC(C)C(=O)NC(CC([O-])=O)C(=O)C[NH2+]CCc1ccccc1	8.33	7.71	0.62
1	CC(C)C(CC(=O)NCCn1c(nc2ccccc12)C)C(=O)NC(CC([O-])=O)C=O	8.37	8.303	0.067
1	CC(C)C(CC(=O)NCCc1cncc2ccccc12)C(=O)NC(CC([O-])=O)C=O	8.39	8.256	0.134
0	CC(C)C(CC(=O)NCCn1ccc2ccccc12)C(=O)NC(CC([O-])=O)C=O	8.4	8.39	0.01
1	CC(NC(=O)C(CCC([O-])=O)NC(=O)C(Cc1ccc(cc1)O)NC(C)=O)C(=O)NC(CC([O-])=O)C(=O)CNS(=O)(=O)CCc1ccccc1	8.4	8.712	-0.312
1	CC(C)C(NC(=O)OCc1ccccc1)C(=O)NC(C)C(=O)NC(CC([O-])=O)C(=O)CNS(=O)(=O)CCc1ccccc1	8.4	8.21	0.19
1	CC(C)C(NC(=O)c1ccc(cc1)Nc1cnc2ccccc2n1)C(=O)NC(C)C(=O)NC(CC([O-])=O)C=O	8.41	8.373	0.037
1	CC(C)C(CC(=O)NCCn1c(nc2ccccc12)-c1cccnc1)C(=O)NC(CC([O-])=O)C=O	8.51	8.298	0.212

1	CC(C)C(NC(=O)OCc1ccccc1)C(=O)NC(C)C(=O)NC(CC([O-])=O)C(=O)COC(=O)Cc1cccc2ccccc12	8.52	8.016	0.504
1	CC(C)C(CC(=O)NCCc1cc(nc2ccccc12)N(C)C)C(=O)NC(CC([O-])=O)C=O	8.55	8.145	0.405
1	CC(C)C(CC(=O)NCCn1cnc2cc(c(cc12)C)C)C(=O)NC(CC([O-])=O)C=O	8.57	8.564	0.006
0	CC(C)C(NC(=O)CCc1ccccc1)C(=O)NC(C)C(=O)NC(CC([O-])=O)C(=O)COC(=O)CCc1ccccc1	8.62	8.252	0.368
1	CCCCCC[NH+](CCCCCC)CC(=O)C(CC([O-])=O)NC(=O)C(CC)N1C=CC=C(NC(=O)c2ccc3ccccc3c2)C1=O	8.64	7.99	0.65
1	CC(C)C(CC(=O)NCCc1cccc2ncccc12)C(=O)NC(CC([O-])=O)C=O	8.74	8.304	0.436
0	CC(C)C(CC(=O)NCCc1ccnc2ccccc12)C(=O)NC(CC([O-])=O)C=O	8.89	8.262	0.628
1	CC(C)C(CC(=O)NCCc1c([nH]c2ccccc12)C)C(=O)NC(CC([O-])=O)C=O	8.9	8.692	0.208
0	CC(C)C(CC(=O)NCCn1c(nc2cc(c(cc12)C)C)C)C(=O)NC(CC([O-])=O)C=O	8.94	8.678	0.262
0	CC(C)C(NC(=O)C(Cc1ccc(cc1)O)NC(C)=O)C(=O)NC(C)C(=O)NC(CC([O-])=O)C(=O)COc1ccccc1	9	8.288	0.712
1	[O-]C(=O)COc1ccc(cc1)C(=O)NC1CCC(=O)N2CCCC(N2C1=O)C(=O)NC(CC([O-])=O)C=O	9	8.098	0.902
1	CC(C)C(NC(=O)OCc1ccccc1)C(=O)NC(C)C(=O)NC(CC([O-])=O)C(=O)COC(=O)CC(c1ccccc1)c1ccccc1	9	8.486	0.514
0	[O-]C(=O)CC(NC(=O)C1CCCN2N1C(=O)C(CCC2=O)NC(=O)c1ccccc1F)C=O	9.02	8.556	0.464
1	CC(C)C(CC(=O)NCCc1c(n(c2ccccc12)C)C)C(=O)NC(CC([O-])=O)C=O	9.07	8.628	0.442
1	CC(C)(C)C(NC(=O)c1ccc(c(c1)Cl)N)C(=O)N1CCCC1C(=O)NC(CC([O-])=O)C=O	9.12	8.565	0.555
1	[O-]C(=O)CC(NC(=O)C1CCCN2N1C(=O)C(CCC2=O)NC(=O)c1nccc2ccccc12)C=O	9.24	8.552	0.688

0 : Test; **1**: Training

Table S2. The values of selected descriptors for all compounds

No.	VE1_X	Chi_Dt	MATS7p	SM13_EA(ed)	SM10_AEA(bo)	DISPm	WiA_Coulomb	TDB03u	TDB07e	TDB08r	RDF110s	Mor10e	nArCO	B05[O-O]	F05[O-O]	RPCG	DLS_02	CATS3D_05_DL
0	5.019	0.16	-0.113	28.311	2.557	8.553	5.674	3.034	7.147	3.892	52.156	-0.297	0	1	5	0.155	0.833	2
1	4.379	0.156	-0.046	28.84	2.782	5.936	5.615	3.057	7.092	3.88	17.253	-0.314	0	1	4	0.163	0.667	2
1	5.76	0.109	0.01	30.366	2.842	3.641	4.849	3.067	6.887	4.005	84.61	-0.064	0	1	4	0.138	0.5	8
1	5.408	0.136	-0.011	28.394	2.624	5.817	5.087	3.049	7.093	4.122	60.388	-1.406	0	1	4	0.144	0.667	5
1	5.296	0.148	-0.067	32.747	2.835	9.423	6.335	3.079	7.126	4.327	27.442	-0.135	0	1	6	0.156	0.667	5
1	5.571	0.133	-0.059	31.088	2.829	7.627	5.093	3.065	7.293	4.172	51.235	-0.543	0	1	6	0.148	0.5	3
0	5.481	0.138	-0.061	31.095	2.83	10.756	4.562	3.054	7.098	3.778	42.826	-0.596	0	1	6	0.152	0.5	7
1	5.499	0.132	0.02	28.354	2.559	7.904	4.775	3.044	6.949	4.095	40.006	-1.948	0	1	4	0.143	0.667	4
1	5.327	0.136	-0.036	28.343	2.54	6.339	4.407	3.04	6.739	3.874	54.537	-1.218	0	1	4	0.163	0.833	2
1	5.117	0.152	-0.087	28.308	2.532	6.221	5.306	3.024	7.11	3.84	38.85	-0.418	0	1	5	0.137	0.667	1
0	5.655	0.117	-0.037	33.349	2.846	5.119	5.881	3.1	6.902	4.264	35.315	-0.333	0	1	6	0.149	0.5	5
1	5.315	0.141	0.001	28.8	2.778	4.455	5.525	3.049	6.893	4.194	46.666	-1.181	0	1	4	0.148	0.667	3
1	5.019	0.16	-0.098	28.311	2.557	7.152	5.366	3.035	6.725	3.511	17.845	-1.159	0	1	5	0.154	0.833	1
1	4.454	0.122	-0.027	31.122	2.838	8.358	4.714	3.064	6.65	3.75	27.007	-0.826	0	1	4	0.153	0.667	4
0	5.829	0.111	-0.078	31.432	3.016	5.369	5.004	3.09	7.332	4.309	54.204	0.045	0	1	6	0.144	0.5	5
1	5.499	0.132	-0.017	28.356	2.543	7.363	4.487	3.04	7.177	4.197	47.988	-0.798	0	1	5	0.143	0.833	2
0	4.384	0.122	-0.05	30.924	2.84	4.952	5.63	3.099	6.809	4.086	46.18	0.728	0	1	4	0.151	0.667	2
1	5.067	0.137	-0.124	30.245	2.951	9.298	4.388	3.047	6.938	3.551	152.672	-1.234	1	1	6	0.108	0.5	5
1	5.067	0.137	-0.138	30.245	2.951	8.957	4.41	3.046	6.809	3.532	69.121	-1.109	1	1	6	0.108	0.5	4
1	5.481	0.138	-0.08	31.095	2.83	5.089	5.343	3.074	7.392	4.25	70.5	-0.613	0	1	6	0.15	0.667	3
1	5.067	0.137	-0.107	30.245	2.951	10.742	4.787	3.057	6.878	3.573	78.59	-0.357	1	1	6	0.117	0.5	4
0	5.025	0.131	-0.076	30.318	2.835	5.945	5.816	3.058	6.681	3.825	2.433	-0.215	0	1	4	0.168	0.833	3
1	4.454	0.122	-0.027	31.122	2.838	8.542	4.763	3.066	6.765	3.79	40.633	-0.373	0	1	4	0.153	0.667	3
1	5.067	0.137	-0.131	30.245	2.951	7.737	4.321	3.04	6.763	3.403	88.044	-0.703	1	1	6	0.111	0.5	6
1	5.748	0.109	0.093	31.252	2.846	5.603	4.698	3.09	6.881	4.289	52.13	-0.763	0	1	5	0.14	0.667	1
0	5.315	0.142	-0.047	28.4	2.62	5.337	5.291	3.055	6.9	4.073	35.309	-1.347	0	1	5	0.147	0.833	2
0	4.454	0.122	-0.04	31.122	2.838	4.502	5.566	3.093	7.195	4.409	39.765	0.633	0	1	4	0.153	0.667	2
1	5.11	0.117	-0.109	30.517	2.966	6.729	3.947	3.052	6.782	3.64	106.922	-1.82	1	1	6	0.103	0.5	8
0	5.76	0.11	-0.088	29.338	2.846	5.42	4.623	3.072	6.855	3.986	70.62	-0.655	0	1	5	0.14	0.667	2
1	5.408	0.137	-0.054	28.36	2.559	3.437	5.069	3.039	7.137	4.032	32.629	-0.241	0	1	5	0.133	0.667	2
1	5.408	0.137	-0.055	28.36	2.559	6.231	5.025	3.05	6.611	3.768	34.791	-0.836	0	1	5	0.146	0.833	3
0	4.829	0.166	0	28.293	2.521	6.923	4.714	3	6.577	3.555	18.65	-0.512	0	1	4	0.125	0.667	4
1	5.748	0.109	-0.036	30.96	2.845	3.951	4.685	3.09	6.87	3.968	78.365	-0.175	0	1	5	0.14	0.667	2

1	5.025	0.132	-0.115	29.317	2.837	6.292	5.697	3.056	6.751	3.835	15.299	-0.538	0	1	4	0.17	0.833	2
0	4.905	0.159	-0.07	29.821	2.829	6.328	6.322	3.045	6.76	3.749	7.05	0.193	0	1	4	0.142	0.667	4
1	5.019	0.16	-0.068	28.311	2.557	14.292	4.209	3.016	7.081	3.637	49.23	-0.582	0	1	5	0.156	0.833	1
0	5.76	0.101	0.05	29.319	2.818	2.574	4.33	3.044	6.776	4.066	40.775	-0.442	0	1	3	0.086	0.5	2
1	5.931	0.095	0.06	29.319	2.818	1.339	3.769	3.037	6.903	4.081	51.188	-0.696	0	1	3	0.083	0.5	4
0	4.792	0.12	0.024	30.388	2.845	4.304	6.005	3.106	7.168	4.52	74.076	-0.873	0	1	5	0.138	0.5	2
1	5.383	0.115	-0.157	30.534	2.838	4.576	5.579	3.079	6.968	3.791	31.345	-0.427	0	1	4	0.133	0.667	3
1	5.846	0.098	0.055	29.319	2.818	2.453	4.214	3.04	6.729	4.015	39.017	-0.308	0	1	3	0.083	0.5	1
0	5.475	0.132	-0.106	30.221	2.837	3.912	5.365	3.063	6.744	3.938	29.141	0.194	0	1	4	0.131	0.667	5
1	5.748	0.109	0.026	30.918	2.846	6.811	4.591	3.088	7.059	4.34	85.656	-0.66	0	1	5	0.14	0.667	2
1	6.01	0.098	0.015	29.588	2.864	2.916	3.93	3.055	6.815	3.913	55.056	-1.852	0	1	3	0.082	0.5	4
0	6.216	0.11	-0.057	30.154	2.919	0.437	3.361	3.037	6.471	3.395	80.651	-1.728	0	1	2	0.083	0.5	9
1	6.365	0.104	-0.081	30.194	2.918	2.701	3.497	3.041	6.549	3.417	119.79	-1.467	0	1	2	0.073	0.333	9
1	5.408	0.137	-0.05	28.36	2.559	6.552	5.376	3.047	6.939	3.972	30.975	-1.016	0	1	5	0.131	0.667	2
1	4.414	0.116	-0.047	33.055	2.846	6.015	6.005	3.101	7.008	4.291	53.073	0.563	0	1	5	0.13	0.667	2
1	6.216	0.108	-0.065	30.137	2.918	1.778	3.415	3.034	6.674	3.57	109.666	-2.178	0	1	2	0.076	0.333	8
0	5.672	0.114	-0.044	29.359	2.845	5.519	4.958	3.075	7.014	4.224	46.129	-1.487	0	1	5	0.141	0.833	2
1	5.365	0.148	0.015	29.472	2.8	10.511	4.805	3.023	7.113	4.085	54.847	-2.073	0	1	9	0.138	0.667	4
1	5.748	0.11	0.026	30.938	2.846	4.205	4.569	3.088	7.046	4.297	60.022	-0.619	0	1	5	0.14	0.667	0
0	6.003	0.113	0.041	30.679	2.641	5.796	4.171	3.056	6.826	4.186	82.815	-1.361	0	1	5	0.09	0.5	7
1	5.207	0.122	-0.104	29.96	2.838	5.848	5.461	3.066	6.828	3.754	23.066	-0.303	0	1	4	0.148	0.833	3
1	4.664	0.119	-0.047	30.443	2.845	7.672	5.771	3.11	7.185	4.448	53.594	-0.823	0	1	5	0.145	0.667	2
1	6.216	0.107	-0.072	30.227	2.918	2.472	3.581	3.035	6.606	3.519	87.602	-0.322	0	1	2	0.08	0.5	8
1	5.931	0.126	-0.046	30.379	2.844	5.571	4.675	3.061	6.764	3.871	92.463	-0.837	0	1	5	0.129	0.5	2
0	5.655	0.123	0.016	30.673	2.641	5.509	4.092	3.041	6.741	3.996	67.945	-1.468	0	1	5	0.097	0.5	6
1	5.748	0.114	0.023	31.126	2.846	5.02	4.797	3.079	6.801	4.015	45.748	-0.47	0	1	5	0.139	0.667	1
1	5.76	0.109	-0.036	30.326	2.846	5.671	5.221	3.071	6.79	3.923	90.373	-1.237	0	1	5	0.125	0.5	2
1	6.01	0.098	0.074	29.588	2.864	5.954	5.056	3.081	7.066	4.524	54.784	-0.403	0	1	3	0.081	0.5	2
1	5.992	0.117	0.02	31.674	2.711	5.555	4.194	3.062	6.668	4.134	46.989	-0.293	0	1	5	0.09	0.5	7
1	6.064	0.113	-0.077	30.135	2.918	7.939	3.081	3.024	6.584	3.433	100.951	-1.975	0	1	2	0.085	0.5	6
1	5.835	0.118	0.053	31.255	2.663	6.65	4.713	3.058	6.843	4.349	44.616	-0.958	0	1	5	0.093	0.5	5
0	4.664	0.119	-0.015	30.443	2.845	3.877	5.301	3.092	7.215	4.442	36.906	-0.633	0	1	7	0.133	0.667	2
0	6.146	0.11	-0.072	30.134	2.918	2.76	3.392	3.031	6.589	3.493	74.5	-1.437	0	1	2	0.082	0.5	6
1	4.664	0.119	-0.032	30.443	2.845	3.93	5.101	3.088	7.212	4.309	57.59	-1.076	0	1	5	0.136	0.667	2
1	6.075	0.11	0.11	30.551	2.84	4.247	4.565	3.064	7.029	4.402	87.275	-0.983	0	1	6	0.112	0.5	6
1	5.908	0.116	0.071	30.629	2.844	3.346	4.861	3.068	6.929	4.311	55.054	-0.418	0	1	5	0.084	0.5	6
0	5.835	0.098	-0.062	30.578	2.838	2.465	4.924	3.069	6.733	4.175	62.707	0.721	0	1	4	0.127	0.5	4
1	5.672	0.113	0.006	30.367	2.845	5.285	4.965	3.078	6.971	4.228	61.518	-0.794	0	1	5	0.141	0.833	2
0	6.233	0.102	0.013	31.251	2.977	2.531	3.878	3.039	6.723	3.797	65.104	0.609	0	1	5	0.079	0.333	4
1	6.302	0.101	0.008	31.252	2.977	5.161	4.391	3.072	6.848	4.21	60.294	-0.325	0	1	5	0.08	0.5	5
1	5.29	0.118	-0.118	30.417	2.838	6.401	5.894	3.072	6.646	3.63	22.287	0.13	0	1	4	0.136	0.667	6
0	5.919	0.098	0.073	29.67	2.89	3.971	3.945	3.055	6.712	3.902	74.714	-1.019	0	1	3	0.083	0.5	3
1	4.207	0.125	-0.091	30.153	2.762	2.9	3.422	3.056	6.699	3.186	35.124	0.597	0	0	0	0.108	1	1
1	6.741	0.097	0.003	31.389	2.989	1.74	3.688	3.057	6.637	3.863	129.579	0.57	0	1	5	0.069	0.333	11

1	4.447	0.115	-0.109	30.626	2.98	6.382	3.668	3.07	6.604	3.129	55.388	0.501	0	0	0	0.104	0.833	2
1	5.557	0.099	-0.059	30.392	2.941	3.623	3.637	3.048	6.659	3.521	86.597	-0.846	0	1	2	0.071	0.5	7
1	4.207	0.125	-0.104	30.153	2.762	1.307	3.309	3.052	6.642	3.139	28.388	0.584	0	0	0	0.109	1	2
1	5.846	0.098	0.062	29.319	2.818	3.355	4.083	3.044	6.689	3.941	69.9	-0.797	0	1	3	0.084	0.5	4
1	4.489	0.116	-0.043	32.083	3.073	11.911	3.722	3.068	6.816	3.267	56.987	1.734	0	0	0	0.116	0.833	2
1	6.359	0.125	-0.005	29.846	2.828	5.594	3.728	3.034	6.8	3.703	129.425	-1.702	0	1	7	0.092	0.5	9
0	6.233	0.099	0.101	30.809	2.966	2.861	4.534	3.082	6.964	4.485	80.55	-1.106	0	1	5	0.08	0.5	4
1	5.383	0.128	0.096	30.707	2.709	7.322	4.96	3.046	6.595	4.036	38.414	-1.276	0	1	5	0.102	0.667	1
0	5.919	0.116	0.013	30.717	2.642	6.436	4.4	3.06	6.755	4.239	118.046	-1.147	0	1	5	0.091	0.5	6
1	6.301	0.114	-0.096	29.75	2.86	2.945	3.156	3.025	6.727	3.486	156.742	-1.9	0	1	6	0.112	0.5	5
1	6.301	0.114	-0.096	29.75	2.86	3.475	3.103	3.025	6.787	3.494	141.466	-2.454	0	1	6	0.112	0.5	6
1	4.43	0.118	-0.082	30.213	2.814	8.867	3.564	3.068	6.801	3.296	56.524	0.812	0	0	0	0.105	0.833	1
1	6.135	0.113	-0.045	30.252	2.922	5.948	2.995	3.031	6.52	3.364	93.012	-1.961	0	1	2	0.086	0.5	7
1	6.37	0.114	-0.04	29.754	2.862	6.583	3.429	3.03	6.617	3.535	92.8	-2.532	0	1	6	0.099	0.5	3
0	4.579	0.099	-0.016	29.668	2.911	6.191	5.458	3.116	7.021	4.839	39.07	-0.925	0	1	3	0.084	0.5	2
1	4.598	0.096	-0.004	29.425	2.838	5.462	5.093	3.117	6.977	4.66	65.76	0.156	0	1	3	0.081	0.5	3
1	6.053	0.107	0.021	31.24	2.976	3.189	4.463	3.065	6.836	3.983	68.549	0.264	0	1	6	0.081	0.5	5
1	5.104	0.156	-0.106	29.373	2.54	9.128	3.969	3.023	6.491	3.221	39.896	-1.813	0	1	4	0.099	0.833	4
1	6.432	0.115	-0.028	31.447	2.974	6.861	3.471	3.057	6.806	3.633	97.875	-2.602	0	1	5	0.066	0.5	5
0	6.221	0.118	-0.02	29.75	2.86	4.705	3.063	3.027	6.597	3.55	159.69	-2.7	0	1	5	0.074	0.5	5
1	6.01	0.098	0.031	29.588	2.864	4.925	4.667	3.081	6.844	4.301	145.476	-0.516	0	1	3	0.082	0.5	4
1	5.015	0.117	-0.12	30.058	2.835	3.572	4.636	3.067	6.549	3.765	85.921	-0.901	0	1	3	0.082	0.5	2
1	6.233	0.098	0.102	31.389	3.062	2.923	4.58	3.084	6.851	4.452	57.151	0.221	0	1	5	0.08	0.5	6
1	4.822	0.087	0.094	31.963	2.937	4.541	4.275	3.071	6.668	4.322	86.113	-0.299	0	1	4	0.073	0.667	4
1	5.433	0.148	0.023	30.282	2.976	9.802	6.23	3.076	6.463	3.66	11.952	-2.437	0	1	5	0.11	0.5	3
0	6.216	0.104	0.029	31.24	2.976	4.093	4.067	3.053	6.788	3.909	96.152	-1.305	0	1	6	0.078	0.5	6
1	6.676	0.096	0.019	31.27	2.976	3.642	3.832	3.05	6.662	3.872	115.531	-1.625	0	1	5	0.068	0.167	13
1	4.366	0.119	-0.046	30.175	2.786	1.471	3.24	3.058	6.701	3.185	35.646	0.139	0	0	0	0.099	1	2
0	5.284	0.145	-0.131	29.392	2.651	8.457	3.777	3.032	6.583	3.271	59.19	-1.95	0	1	4	0.086	0.833	4
1	6.391	0.097	0.12	31.481	3.072	2.623	4.152	3.07	6.856	4.391	91.54	-0.62	0	1	5	0.078	0.5	8
1	6.627	0.095	0.069	31.783	3.096	2.526	4.523	3.081	6.916	4.573	86.638	0.686	0	1	5	0.073	0.5	7
0	6.302	0.101	0.026	31.252	2.977	3.685	3.713	3.047	6.757	3.902	78.403	-1.413	0	1	5	0.079	0.333	2
1	6.755	0.11	0	29.831	2.888	3.694	2.974	3.031	6.367	3.529	157.483	-3.173	0	1	5	0.073	0.5	10
0	6.458	0.099	0.032	31.251	2.977	3.246	3.746	3.055	6.616	3.969	117.907	-0.433	0	1	5	0.079	0.5	9
1	6.458	0.098	0.025	31.274	2.976	3.044	4.287	3.066	6.954	4.247	109.71	-0.717	0	1	6	0.075	0.5	4
1	5.445	0.15	-0.121	29.647	2.834	9.717	4.587	3.025	6.919	3.525	21.639	-1.523	0	1	9	0.137	0.667	3
1	6.216	0.116	-0.031	31.104	2.988	3.87	4.531	3.066	6.687	4.135	125.123	-1.157	0	1	6	0.074	0.5	5
1	5.072	0.113	-0.113	30.071	2.84	4.256	5.242	3.082	6.533	3.827	108.656	-1.429	0	1	3	0.081	0.5	3
0	4.671	0.069	-0.121	35.718	3.528	2.481	2.617	3.096	6.486	3.089	128.899	-0.403	0	0	0	0.085	0.667	4
1	4.671	0.069	-0.121	35.718	3.528	3.18	2.59	3.097	6.48	3.115	85.091	0.539	0	0	0	0.085	0.667	4
1	4.535	0.114	-0.03	30.221	2.853	1.869	3.169	3.065	6.72	3.159	55.774	-0.845	0	0	0	0.089	0.833	2
1	6.216	0.107	-0.07	30.227	2.918	2.042	3.541	3.032	6.613	3.482	73.979	-0.869	0	1	2	0.075	0.333	7
1	6.468	0.097	0.034	31.481	3.072	2.626	4.041	3.065	6.902	4.274	138.885	-0.014	0	1	5	0.075	0.5	8
1	6.233	0.102	0.015	31.251	2.977	2.101	3.976	3.048	6.617	3.86	57.789	-0.821	0	1	5	0.078	0.333	5

1	6.163	0.098	0.091	30.34	2.944	4.746	4.772	3.072	7.065	4.516	72.603	0.383	0	1	5	0.081	0.5	5
1	5.268	0.098	-0.088	30.326	2.937	4.012	4.662	3.081	6.412	3.648	67.699	-0.763	0	1	3	0.077	0.5	2
0	6.312	0.098	0.12	31.473	3.071	2.396	4.351	3.076	6.849	4.39	95.574	-0.877	0	1	5	0.079	0.5	7
1	6.621	0.095	0.088	31.481	3.072	5.633	3.607	3.057	6.755	4.261	137.451	-0.51	0	1	5	0.075	0.5	8
1	6.163	0.098	0.091	31.154	2.984	4.029	4.75	3.072	7.069	4.576	83.544	0.036	0	1	5	0.081	0.5	4
1	6.365	0.101	0.017	31.246	2.977	4.426	3.628	3.04	6.839	3.904	161.788	-0.97	0	1	5	0.073	0.333	6
1	6.375	0.101	0.03	31.24	2.976	6.801	3.648	3.045	6.896	3.969	145.505	-0.893	0	1	6	0.076	0.5	6
0	6.37	0.115	-0.029	29.779	2.876	7.378	3.437	3.052	6.75	3.635	169.431	-2.586	0	1	5	0.07	0.5	4
1	4.207	0.125	-0.054	30.153	2.762	5.371	2.817	3.041	6.631	3.074	40.879	0.053	0	0	0	0.111	1	2
0	5.054	0.097	0.1	32.161	2.729	5.55	4.856	3.053	6.789	4.007	30.877	-1.018	0	1	7	0.119	0.667	4
1	6.676	0.096	0.019	31.27	2.976	2.468	3.779	3.051	6.773	3.97	122.303	-0.611	0	1	5	0.068	0.167	7
1	6.627	0.095	0.082	31.783	3.096	2.098	4.348	3.082	6.919	4.573	141.979	-0.233	0	1	5	0.075	0.5	8
1	6.585	0.112	-0.004	31.176	2.919	5.308	4.094	3.051	7.2	4.053	201.472	-1.365	0	1	8	0.098	0.5	5
0	5.97	0.108	0.029	31.244	2.976	2.089	4.113	3.051	6.487	3.79	67.852	-0.561	0	1	5	0.085	0.333	6
1	5.383	0.137	-0.185	29.732	2.806	6.325	4.013	3.042	6.418	3.009	22.911	-0.941	0	1	5	0.087	0.833	0
1	6.381	0.101	-0.021	31.976	3.103	3.253	4.179	3.072	6.715	4.042	115.585	0.261	0	1	5	0.078	0.5	7
0	4.405	0.13	-0.136	30.607	2.958	2.769	4.572	3.058	6.12	2.921	33.207	-0.825	0	1	4	0.086	0.667	5
1	5.104	0.156	-0.144	29.373	2.54	7.769	4.27	3.016	6.579	3.281	50.386	-1.055	0	1	5	0.088	0.833	2
1	5.891	0.1	-0.105	30.61	2.958	11.05	3.268	3.03	6.415	3.49	53.616	-1.224	0	1	4	0.084	0.333	3
0	6.468	0.097	0.059	31.481	3.072	3.432	3.969	3.065	6.801	4.226	93.996	-0.288	0	1	5	0.077	0.5	8
1	6.003	0.099	0.035	29.539	2.821	4.699	3.747	3.042	6.465	3.522	64.296	-1.941	0	1	3	0.081	0.5	4
1	4.251	0.121	-0.148	30.141	2.741	0.314	3.194	3.052	6.466	3.028	38.741	-0.005	0	0	0	0.107	1	4
0	5.445	0.15	-0.121	29.647	2.834	9.603	4.555	3.024	6.957	3.619	118.814	-2.257	0	1	9	0.137	0.667	3
1	6.233	0.1	0.054	30.997	2.974	3.514	4.488	3.074	6.934	4.364	90.448	-0.17	0	1	5	0.08	0.5	5
0	6.448	0.099	0.016	31.271	2.977	1.706	3.778	3.047	6.833	3.946	123.281	-0.478	0	1	5	0.073	0.333	5
1	6.301	0.114	-0.029	29.75	2.86	6.574	3.282	3.045	6.675	3.655	140.966	-2.59	0	1	5	0.072	0.5	4
0	6.656	0.11	-0.065	30.007	2.88	3.511	3.205	3.028	6.554	3.325	139.69	-1.76	0	1	6	0.068	0.5	16
1	6.702	0.093	0.048	31.501	3.077	1.806	4.163	3.083	6.777	4.43	92.331	-0.6	0	1	5	0.073	0.5	7
1	5.548	0.154	-0.15	29.634	2.716	6.883	4.178	3.033	6.697	3.368	54.864	-1.42	0	1	5	0.083	0.667	0
0	5.852	0.143	-0.069	29.929	2.861	9.269	4.177	3.029	6.548	3.366	84.119	-2.3	0	1	9	0.08	0.5	10
1	6.601	0.096	0.042	31.286	2.977	4.753	3.513	3.047	6.716	3.777	110.34	-0.861	0	1	5	0.072	0.5	7
1	4.424	0.127	-0.114	30.679	2.958	2.674	4.466	3.069	6.517	3.336	69.97	-1.025	0	1	4	0.089	0.833	0
1	6.391	0.101	0.041	31.264	2.977	2.826	4.433	3.074	6.923	4.222	81.068	-0.277	0	1	6	0.073	0.5	4
1	6.233	0.099	0.055	31.499	3.081	3.615	4.54	3.074	6.886	4.363	88.129	-0.422	0	1	5	0.08	0.5	5
1	5.425	0.095	-0.067	32.033	2.978	4.843	3.364	3.057	6.624	3.383	161.5	-0.719	0	1	6	0.086	0.5	5
1	4.754	0.203	-0.019	29.615	2.832	12.811	5.212	3.013	6.574	3.324	48.794	-1.192	0	1	8	0.105	0.833	1
0	6.468	0.1	0.002	31.669	3.091	4.455	3.925	3.058	6.831	4.109	92.799	-0.369	0	1	5	0.076	0.5	6
1	4.405	0.13	-0.117	30.607	2.958	5.914	4.147	3.059	6.599	3.457	39.086	-0.718	0	1	4	0.092	0.833	2
1	5.063	0.101	0.094	32.177	2.831	5.812	5.172	3.06	6.703	3.932	59.761	-0.893	0	1	6	0.087	0.833	2
1	4.421	0.126	-0.146	30.655	2.958	7.282	4.055	3.068	6.668	3.422	32.066	-1.63	0	1	4	0.085	0.667	1
1	6.221	0.118	-0.02	29.75	2.86	4.796	3.194	3.017	6.314	3.286	125.111	-2.02	0	1	5	0.077	0.333	5
1	4.426	0.125	-0.091	31.226	2.958	4.696	4.378	3.068	6.287	3.005	24.367	-0.876	0	1	4	0.086	0.667	4
1	5.383	0.122	-0.087	30.544	2.958	4.883	4.189	3.052	6.353	3.403	56.516	-1.176	0	1	4	0.094	0.833	2
0	4.405	0.13	-0.127	30.607	2.958	5.274	4.304	3.056	6.354	3.118	48.216	0.062	0	1	4	0.092	0.833	4

1	6.926	0.109	-0.02	31.241	2.95	4.364	3.769	3.052	6.93	3.845	248.298	-2.146	0	1	8	0.061	0.333	9
1	6.432	0.114	-0.104	31.274	2.977	5.451	3.881	3.052	6.953	3.561	188.004	-2.32	0	1	8	0.099	0.5	5
1	6.135	0.106	-0.081	30.119	2.934	4.644	4.076	3.051	6.677	3.607	152.676	-1.843	0	1	5	0.069	0.5	5
1	4.572	0.103	-0.117	31.798	2.958	3.51	4.247	3.073	6.33	3.197	52.554	-0.509	0	1	4	0.076	0.5	5
1	6.596	0.1	-0.073	30.709	2.978	5.414	3.564	3.053	6.83	3.671	109.265	-2.31	0	1	7	0.095	0.5	4
1	5.631	0.11	-0.139	30.755	2.958	6.265	3.691	3.061	6.351	3.189	57.215	-1.584	0	1	4	0.083	0.5	1
1	4.44	0.123	-0.136	30.797	2.958	7.657	3.886	3.081	6.449	3.215	28.655	-1.621	0	1	4	0.085	0.667	0
0	6.37	0.114	-0.037	29.754	2.862	7.135	3.34	3.033	6.693	3.552	138.596	-3.89	0	1	6	0.079	0.5	4
1	6.755	0.097	0.041	31.25	2.977	7.892	2.799	3.022	6.538	3.608	261.229	-2.179	0	1	5	0.076	0.5	10
1	5.383	0.122	-0.093	30.544	2.958	4.984	4.205	3.049	6.41	3.422	56.817	-1.528	0	1	4	0.093	0.833	1
0	5.383	0.122	-0.093	30.544	2.958	4.728	4.211	3.051	6.344	3.394	24.289	-1.159	0	1	4	0.093	0.833	1
1	4.426	0.125	-0.074	31.226	2.958	6.606	3.989	3.069	6.301	3.268	47.334	-1.155	0	1	4	0.091	0.833	1
0	4.462	0.119	-0.233	31.352	2.958	7.937	3.695	3.086	6.42	3.06	58.976	-1.612	0	1	4	0.085	0.667	2
0	6.427	0.12	-0.076	30.009	2.883	4.954	3.621	3.037	6.497	3.401	121.035	-3.404	0	1	6	0.065	0.5	12
1	5.015	0.093	0.083	32.179	2.856	2.165	5.011	3.062	6.854	4.233	76.012	-0.969	0	1	6	0.075	0.5	2
1	6.819	0.105	-0.098	30.717	2.978	4.666	3.344	3.046	6.817	3.611	209.157	-3.166	0	1	7	0.091	0.5	3
0	5.221	0.099	0.094	32.227	2.964	4.16	5.552	3.065	6.747	3.859	66.852	-0.412	0	1	6	0.084	0.667	2
1	4.432	0.121	-0.021	32.062	2.958	8.046	3.79	3.084	6.304	3.294	46.523	-1.238	0	1	4	0.091	0.833	1
1	4.711	0.134	-0.19	32.837	3.029	1.388	4.748	3.081	6.414	3.211	29.183	-1.377	0	1	5	0.081	0.667	4
1	5.092	0.087	0.093	32.394	3.107	3.908	4.967	3.071	6.867	4.359	57.568	-0.603	0	1	6	0.082	0.667	3

0: Test; **1**: Training

Table S3. Top 14 natural product hits from the LOTUS database identified by the QSAR model and their predicted pKi values

**LOTUS ID**	**Predicted pKi**
LTS0274950	9.21
LTS0080339	9.16
LTS0091693	9.48
**LTS0070407***	**9.88**
**LTS0016840***	**10.82**
LTS0140410	9.74
LTS0050358	9.17
LTS0170777	9.50
LTS0059426	9.60
**LTS0162325***	**9.93**
**LTS0221286***	**10.03**
LTS0115315	9.16
LTS0199816	13.07
LTS0029227	9.19

*** :** Lead compounds
